# Prediction and Analysis of Skin Cancer Progression using Genomics Profiles of Patients

**DOI:** 10.1038/s41598-019-52134-4

**Published:** 2019-10-31

**Authors:** Sherry Bhalla, Harpreet Kaur, Anjali Dhall, Gajendra P. S. Raghava

**Affiliations:** 1 Center for Computational Biology, Indraprastha Institute of Information Technology, New Delhi, India; 2 Centre for Systems Biology and Bioinformatics, Panjab University, Chandigarh, India; 3 CSIR-Institute of Microbial Technology, Chandigarh, India

**Keywords:** Melanoma, Data processing

## Abstract

The metastatic Skin Cutaneous Melanoma (SKCM) has been associated with diminished survival rates and high mortality rates worldwide. Thus, segregating metastatic melanoma from the primary tumors is crucial to employ an optimal therapeutic strategy for the prolonged survival of patients. The SKCM mRNA, miRNA and methylation data of TCGA is comprehensively analysed to recognize key genomic features that can segregate metastatic and primary tumors. Further, machine learning models have been developed using selected features to distinguish the same. The Support Vector Classification with Weight (SVC-W) model developed using the expression of 17 mRNAs achieved Area under the Receiver Operating Characteristic (AUROC) curve of 0.95 and an accuracy of 89.47% on an independent validation dataset. This study reveals the genes *C7, MMP3, KRT14, LOC642587, CASP7*, *S100A7* and miRNAs hsa-mir-205 and hsa-mir-203b as the key genomic features that may substantially contribute to the oncogenesis of melanoma. Our study also proposes genes *ESM1, NFATC3, C7orf4, CDK14, ZNF827*, and *ZSWIM7* as novel putative markers for cutaneous melanoma metastasis. The major prediction models and analysis modules to predict metastatic and primary tumor samples of SKCM are available from a webserver, CancerSPP (http://webs.iiitd.edu.in/raghava/cancerspp/).

## Introduction

Cancer is one of the major causes of mortality worldwide since the last few decades. According to GLOBOCAN, 2018, 18.1 million new cancer cases and 9.6 million deaths have been estimated worldwide. The melanoma contributes 1.6% of the new cancer cases and 0.6% of deaths due to cancer worldwide^1^. As per the American Cancer Society, there is an estimation of 96,480 melanoma related new cases and 7,230 deaths in 2019 in the US. Melanoma is more prominent in males as compared to females^2^. The malignant transformation of normal human epithelial melanocytes, located within the basement membrane of the skin results in melanoma development. There are several genetic^3^ and environmental factors such as excessive exposure of UV radiations, indoor tanning devices and contacts with certain chemicals like arsenic and hydrocarbons, *etc*. that contribute to melanoma carcinogenesis^4^.

Recently, with the advancement of genomic technologies, there is a huge increment in the generation of big multi-omics data, particularly in the field of cancer^5^, which can be explored for the identification of diagnostic and prognostic cancer biomarkers^6^. The Cancer Genome Atlas (TCGA) is one of the prominent and inclusive repository containing genomic, transcriptomic, epigenetic, proteomics and clinical information of 33 types of cancer^7^. The core study on SKCM done by TCGA has revealed four subtypes of cancer, which include mutant *BRAF*, mutant *RAS*, mutant *NF1*, triple WT (wild-type) based on mutant genes. The triple WT SKCM subtype mainly exhibits *KIT* mutations, focal amplifications and structural rearrangements. Further, it has been observed that the mutational rate of these genes is much higher in melanoma patients than other cancer types of TCGA^8,9^. Interestingly, over 50% of melanoma patients have *BRAF* kinase (*BRAF* proto-oncogene, serine/threonine kinase) mutations^10^. In addition, various studies have demonstrated that the SKCM arises from the anomalies in transcriptomic and epigenetic factors such as expression of mRNAs, miRNAs, the aberration in methylation patterns of CpG islands of genes and histone modifications, which paves the way for the development of potential molecular biomarkers in melanoma^11–23^. In the past, several reports have revealed the potential role of miRNA expression as prognostic biomarkers in cutaneous melanoma. For instance, miR205 and miR29c both act as tumor suppressors and down-regulate the expression of *E2F1*, *E2F5*^24^ and *DNMT*3^15^ genes, respectively. Besides miRNAs, histone methyltransferases also act as crucial players in the progression of melanoma by enhancing the expression of enhancer of zeste homolog 2 (*EZH2*)^25^.

Earlier studies have scrutinized the distinctions between primary melanoma and metastatic melanoma^26–32^. The metastasis mechanism involves several pathways including epithelial-mesenchymal transition (EMT), angiogenesis and invasion. Furthermore, the aggressive stage of melanoma can metastasize to lymph nodes, distinct tissues and organs^33^. Although the survival of cutaneous melanoma patients is affected by various factors, the disease’s early stage diagnosis is one of the most vital parameters with the greatest impact on survival. Different studies have shown the metastasis-free malignant melanoma patients, *i.e*. patients with primary tumor have significantly prolonged survival^34,35^. Evidently, the five-year relative survival rate of melanoma patients is 23%, 64% and 98% for distinct, regional stage and localized tumors, respectively^2^. Hence, the detection of tumor at a localized stage, *i.e*. primary tumor is crucial for patient management and implementation of an appropriate therapeutic strategy for prolonged survival of patients. The genomic and epigenomic biomarkers that can detect primary tumor with high precision might prove to be a boon in this regard and can eventually result in the better outcome of the patients with personalized treatment.

Previously, several stochastic stage wise prediction and classification methods have been developed for diverse cancer types^36,37^. Recently, one study has predicted the metastatic progression score for the assignment of metastatic and primary melanoma based on key miRNA and mRNA expression based putative biomarkers^38^. Although, all the metastatic samples were correctly assigned to a metastatic category based on metastatic progression score. But, the lack of gold standard performance measures like sensitivity, specificity and AUROC, absence of the performance on independent validation dataset and the unavailability of any web-service to analyse new data based on those identified markers are the major lacunae. Hence, the current study is designated to overcome these inadequacies.

In this analysis, we have made an effort to understand the cutaneous skin melanoma progression based on multi-omics layers of data in TCGA that comprises of RNAseq, miRNAseq and methylation expression. Through state-of-the-art machine learning-based feature selection techniques, we have identified genomic signatures that can categorize both primary and metastatic samples with high accuracy. Subsequently, prediction models were developed based on these key identified genomic features using several supervised machine learning techniques that can segregate primary and metastasized SKCM patients.

## Results

**Figure 1:**
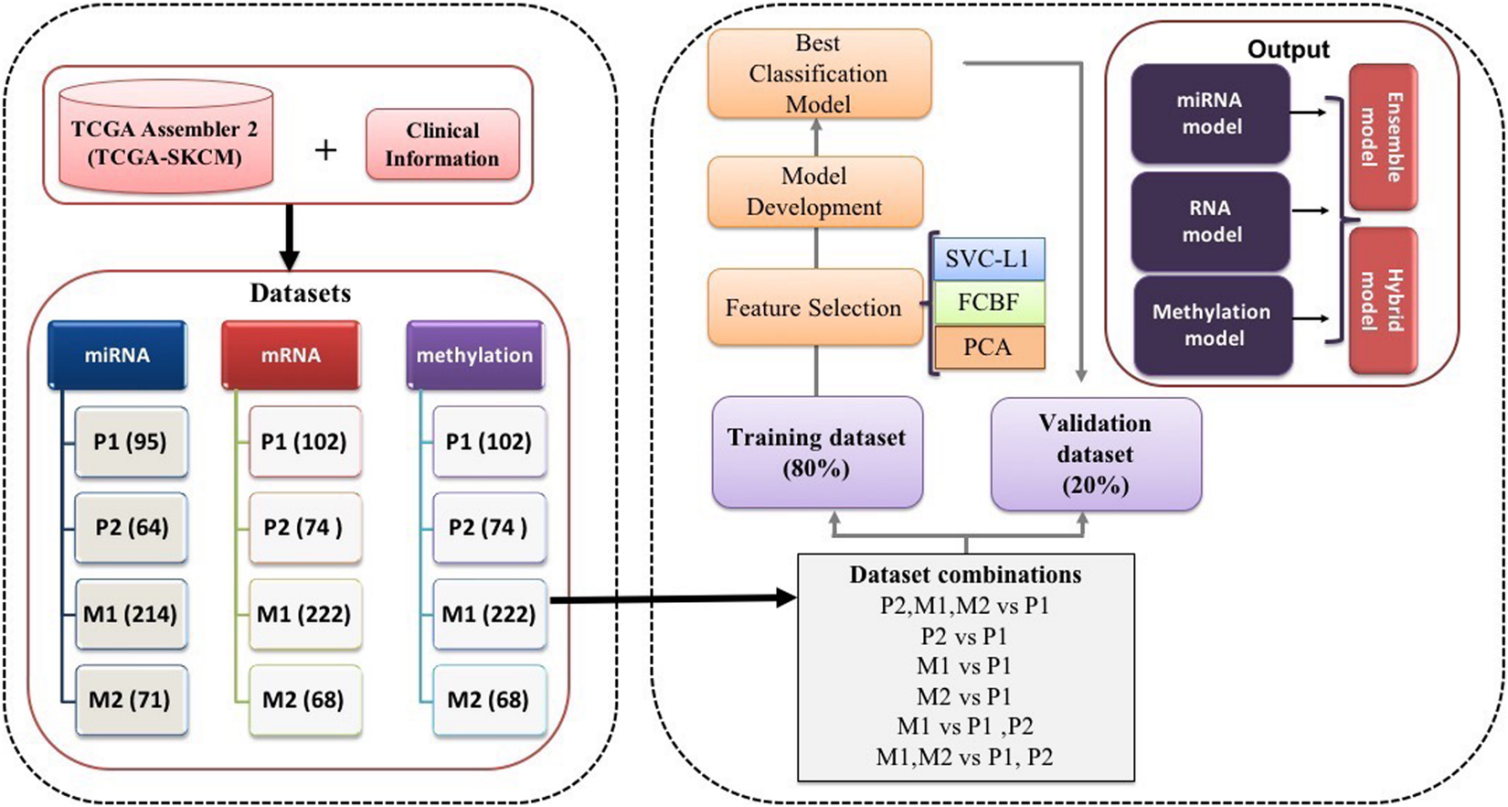
The workflow of the study.

In the current study, we have analysed the RNAseq, miRNAseq, methylation-seq data of SKCM from TCGA for 466, 444, 466 patients, respectively. To mine important genomic and epigenomic features which can discriminate various degree of metastatic tumors (P2, M1 and M2) from the primary tumors (P1), we used well established feature selection methods like WEKA-FCBF^39,40^, Support vector machines with L1 regularization (SVC-L1)^41,42^ and Principal Component Analysis (PCA)^43^. These methods have been previously used in various studies^36,37,44–49^. Subsequently, prediction models have been developed implementing several machine learning techniques like ExtraTrees^50^, KNN, Random forest^51^, Logistic Regression (LR)^52^, Ridge classifier^53^ and SVC - RBF kernel with class weight factor employing scikit package^54^ (described in Methods). The pipeline depicting the workflow of this study is shown in Fig. 1.

### Gene expression based models

**Figure 2:**
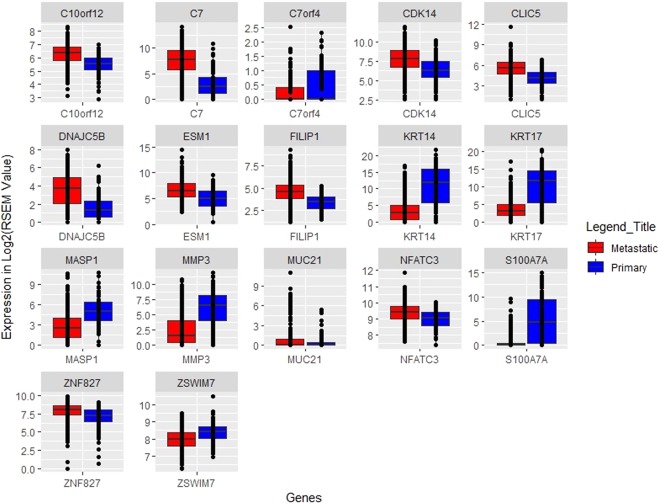
The expression pattern of 17 genes selected using SVC-L1.

With an aim to classify the metastatic and primary tumor samples with high precision, first the RNAseq expression data of 466 patients consisting of 20,502 genes was used to select the relevant features using three feature selection methods; SVC-L1, WEKA-FCBF and PCA. Primarily, we obtained nearly 150 and 17 features using WEKA-FCBF and SVC-L1, respectively. Further, we applied six different machine learning algorithms on the selected features obtained using the above three methods. As shown in Table 1, nearly 92.76% (sensitivity) metastatic tumors and 90.12% (specificity) primary tumors of training dataset and 89.19% (sensitivity) metastatic and 90.48% (specificity) primary tumor samples of validation dataset are correctly identified by SVC-model based on these 17 features (selected by SVC-L1). This model achieved accuracy of 92.18% and 89.47% with AUROC of 0.97 and 0.95 on training and validation dataset, respectively (Table 1). We have selected these above threshold dependent measures based on the threshold of SVM score (decision function in scikit) that gave maximum accuracy along with the minimum difference between sensitivity and specificity (Supplementary Table S1). The boxplot depicting the expression pattern of these 17 features in metastatic and primary tumor samples is shown in Fig. 2.

**Table 1:** Performance measures of 17 mRNA expression based features (selected by SVC-L1 feature selection method) on training and independent validation dataset to classify metastatic from primary tumor samples applying various machine-learning algorithms (classifiers).

Classifiers	Dataset	TP	FP	TN	FN	Sens (%)	Spec (%)	Acc (%)	MCC	AUROC
ETrees	Training	268	12	69	22	92.41	85.19	90.84	0.75	0.95
	Validation	67	5	16	7	90.54	76.19	87.37	0.65	0.94
KNN	Training	269	9	72	21	92.76	88.89	91.91	0.78	0.95
	Validation	66	5	16	8	89.19	76.19	86.32	0.62	0.93
RF	Training	260	8	73	30	89.66	90.12	89.76	0.74	0.96
	Validation	66	2	19	8	89.19	90.48	89.47	0.73	0.95
LR	Training	261	8	73	29	90	90.12	90.03	0.74	0.97
	Validation	65	2	19	9	87.84	90.48	88.42	0.71	0.95
RC	Training	262	9	72	28	90.34	88.89	90.03	0.74	0.96
	Validation	65	2	19	9	87.84	90.48	88.42	0.71	0.95
SVC-W	Training	269	8	73	21	92.76	90.12	92.18	0.79	0.97
	Validation	66	2	19	8	89.19	90.48	89.47	0.73	0.95

Etrees: Extra Trees Classifier; KNN: K-Nearest Neighbors Classifier; RF: Random Forest; LR: Logistic Regression; RC: Ridge Classifier; SVC-W: Support Vector Classification with weight factor; TP: True positive; FP: False Positive; TN: True Negative; FN: False Negative; Sens: Sensitivity; Spec: Specificity; Acc: Accuracy; MCC: Matthews Correlation Coefficient; AUROC: Area under the Receiver Operating Characteristic.

Interestingly, a model based on 150 features selected using WEKA-FCBF also attained almost similar performance (Supplementary Table S2). Further, we also selected 32 Principal Component features using Principal Component Analysis (PCA), each of which explains at least 1% of the variance in the data. Logistic Regression (LR) based prediction model performed best, classifying metastatic and primary samples with 92.96% sensitivity, 76.19% specificity, 89.13% accuracy with 0.91 AUROC on validation dataset (Supplementary Table S3). As the models based on features selected by SVC-L1 have smaller number of features and higher performance as compare to the models based on features selected by WEKA-FCBF and PCA, respectively. We considered and reported the model based on 17 features as best expression-based classification model to distinguish metastatic and primary tumor samples of SKCM.

The enrichment analysis of the 17 features shows the biological role of the mRNA signature in melanoma carcinogenesis. Out of 17 genes, *C7* and *MASP1* are involved in Complement system activation (adjusted p-value < 0.05), while *KRT17* and *KRT14* are part of intermediate filament component (adjusted p-value < 0.05). It has been shown that metastatic cancer cells use actin bundles to disrupt from a primary tumour and invade the surrounding tissue. After travelling in the vasculature or lymphatic system, they exit into a new niche and form a new tumour^55^.

As we analysed the new tumour event (NTE) clinical file of SKCM patients, we observed that 16 patients with primary tumor have been shown to be in distant metastasis with new tumour events. Therefore, we removed these 16 samples from the dataset and again developed the classification model. There was a marginal increase of MCC from 0.73 to 0.77 and alike AUROC on validation dataset (Supplementary Table S4).

**Figure 3:**
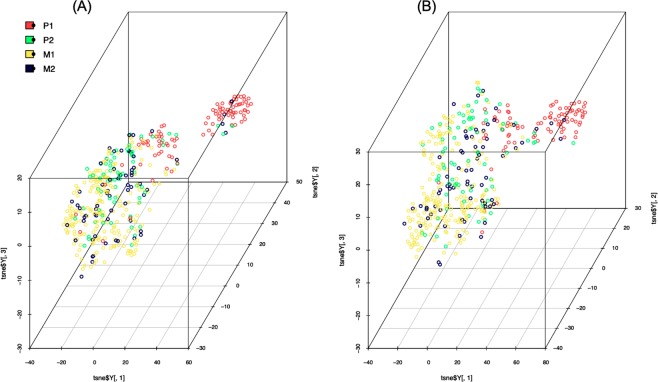
The scatterplot3D view of tSNE dimension reduction of 17 selected features: (**A**) distribution of P1, P2, M1 and M2 samples; (**B**) distribution of P1, P2, M1 and M2 samples after removing 16 primary tumor samples (observed as distant metastatic in NTE file).

From the above analysis, we have observed that 17 mRNA expression-based features are performing reasonably well in classifying metastatic and primary tumor samples. Further, we visualised the samples based on 17 mRNA expression features using t-SNE (t-Distributed Stochastic Neighbour Embedding) implemented using the Rtsne^56^ and scatterplot3D^57^ packages in R. The substantial number of P2 samples differ from P1, but some of them merge/co-clustered with P1, which is quite expected as P1 progresses to P2 (Supplementary Fig. S1(A)). The t-SNE analysis shows a clear distinction between P1 and M1 (Supplementary Fig. S1(B)) with some of the primary samples going extreme into the boundaries of M1. Surprisingly the distant metastatic samples are quite widely distributed in comparison to Primary tumors as shown in Supplementary Fig. S1(C). Next, Supplementary Fig. S1(D) presents the P1tumors in contrast to P2 from M1 tumors. Here P1 tumors looks separated from P2 and M1 whereas P2 and M1 tumors are amalgamated. The Fig. 3(A) shows that primary tumor (P1) samples form the separate cluster (red colour) in comparison to different states of metastasisfor all the samples and Fig. 3(B) shows all the four classes after removing16 primary samples of NTE. This analysis prompt us to further develop specific prediction models for classifying each state of metastasis from primary samples.

### Discrimination between Primary and sub-categories (or various states) of metastasis

#### Intra-lymphatic tumors v/s primary tumors

**Figure 4:**
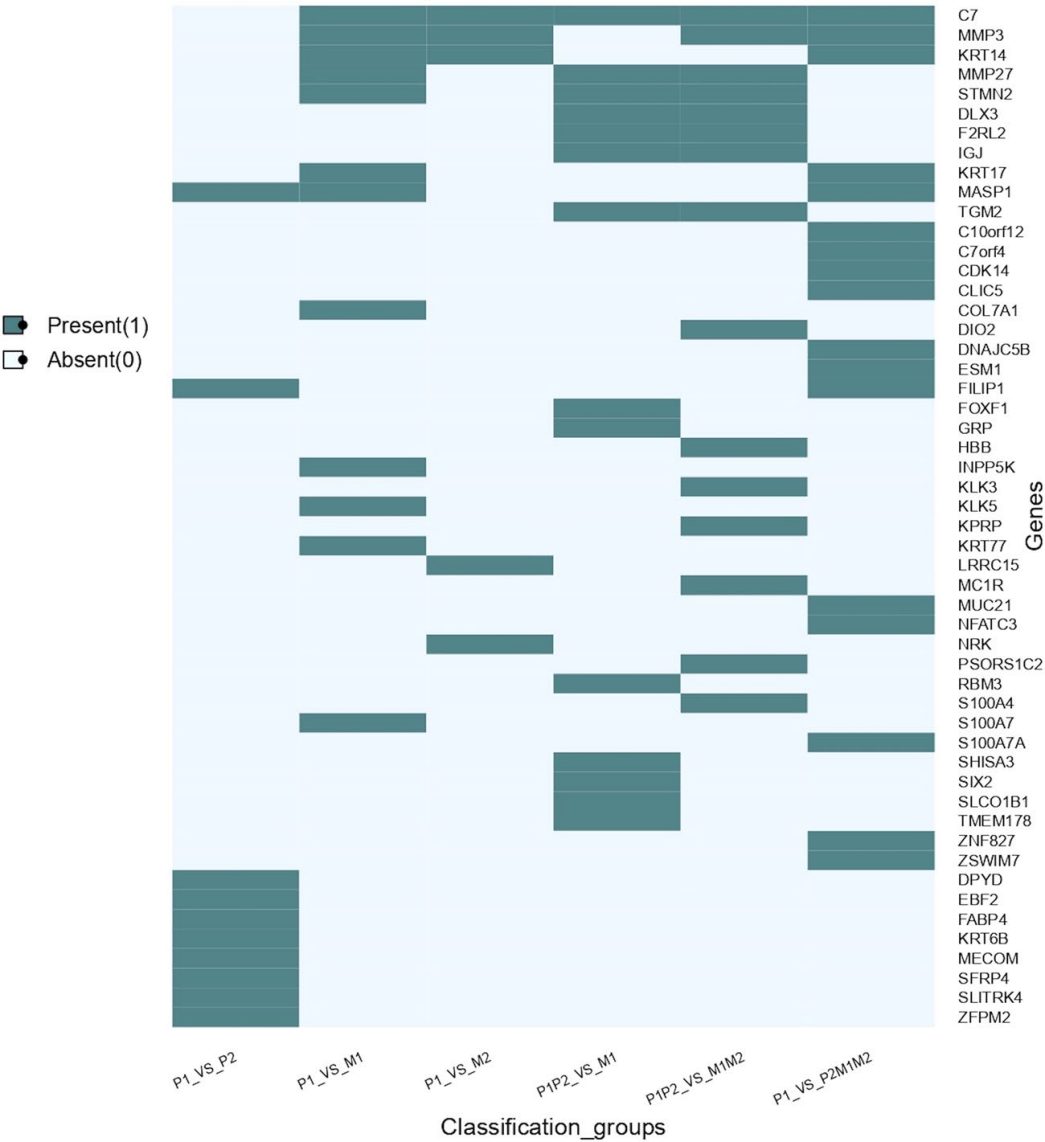
The presence and absence of various features in different gene signatures developed for segregating metastatic samples from primary samples.

The primary tumors (P1) are localised lesions and P2 includes the samples with-in-transit metastasis and satellite metastasis which represent intra-lymphatic tumour. At this stage, tumor has not still spread to lymphatic nodes. We selected 10 features using SVC-L1 (as in the above models, RNAseq data selected the appropriate number of features using SVC-L1) and the results of classification models on these 10 features show that it is difficult to classify the samples with intra-lymphatic tumour (P2) from the primary tumour (P1). The KNN-based model correctly identified 84.75% (Sensitivity) of metastatic and 93.83% (Specificity) of primary tumor patients of training data with MCC of 0.79 and AUROC of 0.96. On validation data, this model identified 73.33% (Sensitivity) of metastatic and 85.71% (Specificity) primary patients correctly with MCC 0.60 and AUROC of 0.84 (Supplementary Table S5). The selected ten features are shown in the Lane 1 of heatmap (Fig. 4).

### Lymphatic tumors v/s primary tumors

Further, we tried to classify tumors that invaded lymphatic nodes (M1) from the primary tumors (P1). Our analysis shows that these tumors can be classified with high precision. The SVC-W based model using mRNA expression of 12 genes (Lane 2 of Fig. 4), selected using SVC-L1 feature selection method distinguished samples with good sensitivity of 97.74%, specificity 91.36% and MCC of 0.90 and AUROC of 0.98 on training data. We also observed the good sensitivity of 95.56% and specificity of 90.48% along with MCC of 0.86 and AUROC of 0.94 on the validation dataset. This indicates that once the tumour has reached the lymph nodes, there is substantial variation in the expression of genes associated with metastasis in comparison to the primary or localized tumor (Table 2).

**Table 2:** Performance measures of 12 mRNA expression features (selected using SVC-L1 feature selection method) to discriminate M1 from P1 on training and independent validation dataset by applying various machine-learning algorithms.

Classifier	Dataset	TP	FP	TN	FN	Sens (%)	Spec (%)	Acc (%)	MCC	AUROC
**ETrees**	Training	170	10	71	7	96.05	87.65	93.41	0.85	0.96
	Validation	44	3	18	1	97.78	85.71	93.94	0.86	0.91
**KNN**	Training	175	10	71	2	98.87	87.65	95.35	0.89	0.95
	Validation	43	4	17	2	95.56	80.95	90.91	0.79	0.92
**RF**	Training	155	7	74	22	87.57	91.36	88.76	0.76	0.96
	Validation	37	2	19	8	82.22	90.48	84.85	0.69	0.93
**LR**	Training	174	8	73	3	98.31	90.12	95.74	0.9	0.98
	Validation	43	2	19	2	95.56	90.48	93.94	0.86	0.93
**RC**	Training	175	10	71	2	98.87	87.65	95.35	0.89	0.97
	Validation	44	3	18	1	97.78	85.71	93.94	0.86	0.95
**SVC-W**	Training	173	7	74	4	97.74	91.36	95.74	0.9	0.98
	Validation	43	2	19	2	95.56	90.48	93.94	0.86	0.94

Etrees: Extra Trees Classifier; KNN: K-Nearest Neighbors Classifier; RF: Random Forest; LR: Logistic Regression; RC: Ridge Classifier; SVC-W: Support Vector Classification with weight factor; TP: True positive; FP: False Positive; TN: True Negative; FN: False Negative; Sens: Sensitivity; Spec: Specificity; Acc: Accuracy; MCC: Matthews Correlation Coefficient; AUROC: Area under the Receiver Operating Characteristic.

#### Distant metastatic tumors v/s primary tumors

Next, we tried to classify the distant metastatic tumors (M2) from primary tumors (P1). Surprisingly the classification of these two groups of samples is not as good as lymphatic node v/s primary on 5 features (Lane 3 of Fig. 4). The KNN model correctly classified 87.04% (Sensitivity) distant metastatic samples and 92.59% (Specificity) primary samples with MCC of 0.80 and AUROC of 0.92 on training data. This model classified 78.57% (Sensitivity) distant metastatic samples and 85.71% (Specificity) primary samples correctly with MCC of 0.64 and AUROC of 0.81 on validation dataset (Supplementary Table S6).

#### Regional v/s lymphatic tumors

To differentiate between the tumors which have spread to lymph nodes (M1) and regional tumors, we combined primary (P1) and in transit and satellite tumors (P2). The LR model using 14 features (Lane 4 of Fig. 4) selected by SVC-L1, achieved the sensitivity of 92.09% and specificity of 90% with MCC of 0.82 and AUROC of 0.96 on training data and the sensitivity of 93.33% and specificity of 83.33% with MCC of 0.78 and AUROC of 0.89 on validation dataset (Supplementary Table S7).

#### Metastatic tumors v/s regional or primary tumor

Further, we developed the model to categorize the tumors which spread to lymph nodes or metastasized (M1 and M2) from the tumors which were localized (P1 and P2). The Logistic Regression (LR) model based on 15 features (Lane 5 of Fig. 4) correctly classified 89.61% metastatic samples of training dataset with MCC of 0.74 and AUROC of 0.93. On validation dataset 81.36% metastatic sample and 80.56% of primary samples are correctly predicted with MCC of 0.61 and AUROC of 0.90 (Supplementary Table S8). The Lane 6 of Fig. 4 shows the 17 mRNA signature that has performed best out of all the combinations.

### miRNA expression based models

Next, we have explored miRNA expression to elucidate its role in the progression of metastasis in SKCM. The number of miRNA features selected by WEKA-FCBF and SVC-L1 is 32 and 5 features, respectively. The SVC-W model based on 32 miRNAs attained the maximum performance with MCC of 0.69 and AUROC of 0.94 on training dataset and MCC of 0.66 and AUROC of 0.89 on the validation dataset. Further, nearly 86.33% (sensitivity) metastatic samples and 90.79% (specificity) primary samples of training dataset and 90.14% metastatic samples and 78.95% primary samples of validation dataset were correctly predicted (Table 3). The mean expression pattern of these 32 miRNA in primary and metastatic samples is represented in Supplementary Fig. S2. The Logistic Regression model based on the 5 miRNAs (feature selected by SVC-L1 method), achieved maximum MCC of 0.62 and AUROC of 0.93 on training dataset and MCC of 0.59 and AUROC of 0.87 on the validation dataset. This model correctly predicted 86.69% metastatic samples and 81.58% primary tumor samples of training dataset and 83.1% metastatic samples and 84.21% primary samples of validation dataset (Table 4). These 5 miRNAs include hsa-mir-205, hsa-mir-218.2, hsa-mir-513a.1, hsa-mir-675 and hsa-mir-7974. Here also, we filtered 43 Principal Component features employing Principal Component Analysis (PCA), each of them exhibits at least 1% variance of the data. The prediction model based on these features using Ridge Classifier method categorized metastatic and primary samples with an accuracy of 81.2% and 81.52% and AUROC 0.88 and AUROC 0.86 of training and validation datasets, respectively (Supplementary Table S9).

**Table 3:** Performance measures of 32 miRNA expression features (selected by WEKA-FCBF feature selection method) on training and independent validation to classify metastatic from primary samples dataset by applying various machine-learning algorithms.

Classifier	Dataset	TP	FP	TN	FN	Sens (%)	Spec (%)	Acc (%)	MCC	AUROC
ETrees	Training	250	14	62	28	89.93	81.58	88.14	0.67	0.92
	Validation	63	5	14	8	88.73	73.68	85.56	0.59	0.88
KNN	Training	234	12	64	44	84.17	84.21	84.18	0.61	0.91
	Validation	60	3	16	11	84.51	84.21	84.44	0.61	0.89
RF	Training	232	8	68	46	83.45	89.47	84.75	0.64	0.97
	Validation	63	3	16	8	88.73	84.21	87.78	0.67	0.95
LR	Training	241	14	62	37	86.69	81.58	85.59	0.62	0.93
	Validation	59	3	16	12	83.1	84.21	83.33	0.59	0.87
RC	Training	245	10	66	33	88.13	86.84	87.85	0.69	0.94
	Validation	62	4	15	9	87.32	78.95	85.56	0.61	0.89
SVC-W	Training	240	7	69	38	86.33	90.79	87.29	0.69	0.94
	Validation	64	4	15	7	90.14	78.95	87.78	0.66	0.89

Etrees: Extra Trees Classifier; KNN: K-Nearest Neighbors Classifier; RF: Random Forest; LR: Logistic Regression; RC: Ridge Classifier; SVC-W: Support Vector Classification with weight factor; TP: True positive; FP: False Positive; TN: True Negative; FN: False Negative; Sens: Sensitivity; Spec: Specificity; Acc: Accuracy; MCC: Matthews Correlation Coefficient; AUROC: Area under the Receiver Operating Characteristic

**Table 4:** Performance measures of 5 miRNA expression features (selected by SVC-L1 feature selection method) on training and independent validation dataset to classify metastatic from primary samples by applying various machine-learning algorithms.

Classifier	Dataset	TP	FP	TN	FN	Sens (%)	Spec (%)	Acc (%)	MCC	AUROC
ETrees	Training	227	18	58	51	81.65	76.32	80.51	0.52	0.88
	Validation	59	3	16	12	83.1	84.21	83.33	0.59	0.86
KNN	Training	226	15	61	52	81.29	80.26	81.07	0.54	0.88
	Validation	57	3	16	14	80.28	84.21	81.11	0.56	0.84
RF	Training	229	18	58	49	82.37	76.32	81.07	0.52	0.9
	Validation	56	3	16	15	78.87	84.21	80	0.54	0.88
LR	Training	241	14	62	37	86.69	81.58	85.59	0.62	0.93
	Validation	59	3	16	12	83.1	84.21	83.33	0.59	0.87
RC	Training	245	14	62	33	88.13	81.58	86.72	0.65	0.93
	Validation	58	3	16	13	81.69	84.21	82.22	0.58	0.88
SVC-W	Training	231	12	64	47	83.09	84.21	83.33	0.60	0.93
	Validation	54	3	16	17	76.06	84.21	77.78	0.51	0.87

Etrees: Extra Trees Classifier; KNN: K-Nearest Neighbors Classifier; RF: Random Forest; LR: Logistic Regression; RC: Ridge Classifier; SVC-W: Support Vector Classification with weight factor; TP: True positive; FP: False Positive; TN: True Negative; FN: False Negative; Sens: Sensitivity; Spec: Specificity; Acc: Accuracy; MCC: Matthews Correlation Coefficient; AUROC: Area under the Receiver Operating Characteristic.

Among the miRNA signatures, hsa-mir-205 targets various genes (identified from miRTarBase^58^) such as *ZEB2, ZEB1, ERBB3, PRKC, ERBB2, E2F1, BCL2, ITGA5, VEGFA, AR, SMAD4, EGLN2, LAMC1, VEGFA, SMAD1, SRC, VEGFA, DDX5* and *YES1*, *etc*. Gene enrichment analysis have shown that these genes are significantly enriched (adjusted p-value < 0.05) in various cell growth promoting and oncogenesis associated pathways including transcriptional misregulation, TGF-beta signaling, wnt signaling, PDGF signaling, EGFR signaling, PI3K signaling, p53 signaling, ErbB signaling, VEGF signaling, cell cycle, hypoxia and angiogenesis, apoptosis processes, *etc*. This analysis signifies the role of hsa-mir-205 as tumor suppressor in melanoma development as it gets downregulated with the progression of metastatic melanoma.

### Methylation based model

To ascertain the role of epigenetics in distinguishing metastatic from primary tumors, we took average methylation beta values for each gene as described in methods. Firstly, 38 and 2 features were selected using WEKA-FCBF and SVC-L1, respectively. Subsequently, classification models were developed using 38 features, and it can be observed in Table 5 that average methylation values are not very good predictors for distinguishing metastatic and primary tumor samples as compared to gene and miRNA expression. For instance, the LR model based on these 38 features achieved maximum performance, able to discriminate them with maximum MCC of 0.48 and 0.44 on training and validation dataset, respectively. It correctly predicted only 76.47% metastatic samples and 79.27% primary tumor samples of training dataset and 78.38% of metastatic samples and 71.43% primary tumor samples of validation dataset (Table 5). Further, 25 Principal Component features from methylation data filtered implementing PCA employing similar criteria like of mRNA and miRNA. SVC-W model based on these features is the best performer that attained an accuracy of 73.22% and 68.48% and AUROC 0.79 and AUROC 0.70 for segregation of tumor samples of training and validation datasets, respectively (Supplementary Table S10).

**Table 5:** Performance measures of 38 features or average methylation of genes (features selected using WEKA-FCBF feature selection method) on training and independent validation dataset to classify metastatic from primary samples by applying various machine-learning algorithms.

Classifier	Dataset	TP	FP	TN	FN	Sens (%)	Spec (%)	Acc (%)	MCC	AUROC
ETrees	Training	248	17	65	41	85.81	79.27	84.37	0.6	0.89
	Validation	65	8	13	9	87.84	61.9	82.11	0.49	0.87
KNN	Training	224	19	63	65	77.51	76.83	77.36	0.47	0.83
	Validation	54	6	15	20	72.97	71.43	72.63	0.38	0.82
RF	Training	255	23	59	34	88.24	71.95	84.64	0.58	0.92
	Validation	65	9	12	9	87.84	57.14	81.05	0.45	0.87
LR	Training	221	17	65	68	76.47	79.27	77.09	0.48	0.84
	Validation	58	6	15	16	78.38	71.43	76.84	0.44	0.85
RC	Training	239	17	65	50	82.7	79.27	81.94	0.56	0.88
	Validation	62	8	13	12	83.78	61.9	78.95	0.43	0.83
SVC-W	Training	221	19	63	68	76.47	76.83	76.55	0.46	0.82
	Validation	58	6	15	16	78.38	71.43	76.84	0.44	0.91

Etrees: Extra Trees Classifier; KNN: K-Nearest Neighbors Classifier; RF: Random Forest; LR: Logistic Regression; RC: Ridge Classifier; SVC-W: Support Vector Classification with weight factor; TP: True positive; FP: False Positive; TN: True Negative; FN: False Negative; Sens: Sensitivity; Spec: Specificity; Acc: Accuracy; MCC: Matthews Correlation Coefficient; AUROC: Area under the Receiver Operating Characteristic.

### Ensemble model

Next, in order to compile information from individual models developed using all the three types of genomic features, we developed an ensemble method. In the ensemble method, prediction score from each model *i.e*. mRNA, miRNA and methylation were provided as input features to SVC. This model attained MCC of 0.73 along with AUROC of 0.97 and 0.71 MCC along with 0.93 AUROC on training and validation dataset, respectively (Table 6).

**Table 6:** Performance measures of RNAseq, miRNAseq and methylation-seq ensemble features on training and independent validation dataset to classify metastatic from primary samples by applying SVC.

Dataset	TP	FP	TN	FN	Sens (%)	Spec (%)	Acc (%)	MCC	AUROC
Training	244	5	71	34	87.77	93.42	88.98	0.73	0.97
Validation	60	1	18	10	85.71	94.74	87.64	0.71	0.93

Etrees: Extra Trees Classifier; KNN: K-Nearest Neighbors Classifier; RF: Random Forest; LR: Logistic Regression; RC: Ridge Classifier; SVC-W: Support Vector Classification with weight factor; TP: True positive; FP: False Positive; TN: True Negative; FN: False Negative; Sens: Sensitivity; Spec: Specificity; Acc: Accuracy; MCC: Matthews Correlation Coefficient; AUROC: Area under the Receiver Operating Characteristic.

### Combo models

As from the above analysis, we observe that 17 mRNAs and miRNA hsa-mir-205 have performed best for discriminating primary and metastatic tumours. Therefore, we combined them and developed models using various machine learning algorithms (Supplementary Table S11). The performance of this hybrid model is almost similar to the model based on 17 mRNA features with a marginal increase in specificity (Table 1).

Additionally, with an aim to extract information from all the three types of genomic features, i.e. RNAseq, miRNAseq and methylation-seq data, and develop multi-omics model, we combined all the features of three types of data by normalizing them using Min-Max normalization (see Methods). We used SVC-L1 method here as this feature selection method has shown reasonably higher performance with a minimum number of features in comparison to WEKA-FCBF and PCA in the previous analyses. The 20 features (Supplementary Table S12) selected by SVC-L1 method include 14 mRNA (genes), 1 miRNA and 5 methylation genes.

Subsequently, various prediction models developed based on these features employing different machine learning techniques. The performance of the most of the prediction models based on these features is in a similar range as of the performance of models based on 17 mRNA expression features for both on training and validation datasets, respectively (Supplementary Table S13).

### Single feature-based classification model using mRNA and miRNA expression

Here, our goal is to develop single feature-based classification models that rank each gene and miRNA for its contribution to distinguish primary and metastatic tumor using threshold-based approach which was implemented in our previous studies for ranking of the genes^36^. In threshold-based model, a sample is classified as metastatic if the log2 RNA-Seq by Expectation Maximization (RSEM) value of the feature (if feature is upregulated in metastatic) is higher than a threshold value, otherwise, it is classified as a primary sample. In these models, the threshold is varied incrementally from minimum to maximum RSEM value. Finally, that threshold is selected, which have the maximum AUROC in classifying metastatic and primary tumor samples. Consequently all the mRNA and miRNA sites are ranked on the basis of maximum AUROC and MCC with the minimum difference in sensitivity and specificity to assess the ability of each feature to classify metastatic and primary samples (Supplementary Table S14). Table S14 represents 20 mRNAs and 2 miRNA that can distinguish two types of samples with high precision.

The hsa-mir-205 and hsa-mir-203b are the top 2 miRNAs that can classify the metastatic and primary tumor samples with AUROC 0.83 and 0.75 at thresholds 4.3 and 1 (log2 RSEM values), respectively. Both of these miRNAs are downregulated in metastatic samples, which indicates their potential role as tumor suppressors. The *C7*, *S100A7, LOC642587, CASP14* and *MMP3* are among the top 5 mRNA expression features that can discriminate metastatic and primary tumor samples with AUROC 0.81, 0.78, 0.77, 0.77 and 0.77 at thresholds 4.3, 3.1, 0.9, 0.9 and 3.7 (log2 RSEM values), respectively. Among them, *C7* is upregulated in metastatic samples, while rest of the observed genes are downregulated in metastatic samples.

### Discriminating the early and late stage Primary SKCM tumors

We further subdivided the heterogenous P1 subgroup according to the SKCM tumor stage. Of the total 103 samples of primary SKCM, the tumor stage information is available for 98 patients with gene expression data and for 96 patients with miRNA expression data.

We have used both WEKA-FCBF and SVC-L1 based feature selection method (described in methods) to extract the important gene expression based features which could discriminate the early stage and late stage primary tumors. The WEKA-FCBF based method resulted in fewer features and better performance. Due to availability of a lesser number of samples (less than 100) we have used leave-one-out cross-validation technique to develop the prediction model using selected 37 (Supplementary Fig. S3) mRNA-based (WEKA-FCBF selected features) expression features. The random forest-based method performed best with a sensitivity of 95.52% and specificity of 83.87% with MCC of 0.81 (Table 7). Many of the genes in this signature have been already shown to be associated with melanoma.

**Table 7:** Performance measures of 37 mRNA expression features (selected using SVC-L1 feature selection method) to discriminate early from late stage primary tumors using leave one out cross validation by applying various machine-learning algorithms.

Classifier	TP	FP	TN	FN	Sen (%)	Spec (%)	Acc (%)	MCC	AUROC
Etrees	58	3	28	9	86.57	90.32	87.76	0.74	0.96
KNN	59	16	15	8	88.06	48.39	75.51	0.4	0.78
RF	64	5	26	3	95.52	83.87	91.84	0.81	0.96
LR	54	5	26	13	80.6	83.87	81.63	0.61	0.87
RC	53	8	23	14	79.1	74.19	77.55	0.51	0.83
SVC-W	62	9	22	5	92.54	70.97	85.71	0.66	0.88

Etrees: Extra Trees Classifier; KNN: K-Nearest Neighbors Classifier; RF: Random Forest; LR: Logistic Regression; RC: Ridge Classifier; SVC-W: Support Vector Classification with weight factor; TP: True positive; FP: False Positive; TN: True Negative; FN: False Negative; Sens: Sensitivity; Spec: Specificity; Acc: Accuracy; MCC: Matthews Correlation Coefficient; AUROC: Area under the Receiver Operating Characteristic.

One of the genes in the signature, *HSF1* has been already shown to be associated with early stage melanoma and has been shown to drive metastasis^59^. Another gene is *CDC37*, which is observed to be an essential gene to maintain the role of proteins that interact with protein kinases in melanoma. Furthermore, *RPS27* has been reported to have mutations in untranslated region and shown to have an impact in the progression of melanoma^60^. We could not find any of genes in this signature that is common with the genes that segregate primary and several forms of metastatic melanomas. This points out the heterogeneous nature of the primary melanomas itself.

Next, miRNA expression was explored to distinguish between the early stage and late stage primary SKCM samples. Using 32 miRNA (Supplementary Fig. S4) features selected by SVC-L1 feature selection method, KNN model is the top performer with balanced sensitivity of 91.8% and specificity of 93.33% with AUROC of 0.96 (Table 8). Of the 32 miRNAs, earlier few have been shown to be associated with melanoma, and many others have observed to be regulated in other cancers. For instance, hsa-mir-198 has been manifested to inhibit invasion of melanoma cells previously and has been downregulated in late stage as compared to an early stage in our analysis^61^. The expression of hsa-mir-219 has been shown to be downregulated in malignant melanoma (consistent with our analysis) and has also exhibited to be an important therapeutic target in melanoma^62^. Other miRNAs such as has-let-7f-1 has been implicated in lung cancer and renal cancer^63,64^, while hsa-mir-219a-1 in renal cancer^65^.

**Table 8:** Performance measures of 32 miRNA expression features (selected using SVC-L1 feature selection method) to discriminate early from late stage primary tumors using leave one out cross validation by applying various machine-learning algorithms.

Classifier	TP	FP	TN	FN	Sen(%)	Spec(%)	Acc(%)	MCC	AUROC
Etrees	52	3	27	9	85.25	90	86.81	0.72	0.93
KNN	56	2	28	5	91.8	93.33	92.31	0.83	0.96
RF	48	3	27	13	78.69	90	82.42	0.65	0.92
LR	55	0	30	6	90.16	100	93.41	0.87	0.99
RC	60	2	28	1	98.36	93.33	96.7	0.93	0.99
SVC-W	59	0	30	2	96.72	100	97.8	0.95	0.99

Etrees: Extra Trees Classifier; KNN: K-Nearest Neighbors Classifier; RF: Random Forest; LR: Logistic Regression; RC: Ridge Classifier; SVC-W: Support Vector Classification with weight factor; TP: True positive; FP: False Positive; TN: True Negative; FN: False Negative; Sens: Sensitivity; Spec: Specificity; Acc: Accuracy; MCC: Matthews Correlation Coefficient; AUROC: Area under the Receiver Operating Characteristic.

### Web server implementation

To contribute the scientific community, we developed a web server, CancerSPP (Skin Cancer Progression Prediction). CancerSPP is designed for the prediction and analysis of metastatic and primary tumor of SKCM from RNAseq, miRNA and methylation expression data. The web server has two modules; Prediction module and Data analysis module.

#### Prediction module

This module permits the users to predict different states of metastatic samples and primary tumor samples *i.e*. Intra-lymphatic tumors ((P2) v/s Primary tumor (P1), Lymphatic tumors (M1) v/s Primary tumors (P1), Distant Metastatic tumors (M2) v/s Primary tumors (P1), Regional (P1P2) v/s Lymphatic tumors (M1) and Metastatic tumors (M1M2) v/s Regional tumor (P1P2) utilizing RSEM expression quantification values of signature genes. The user needs to submit the RSEM value of signature genes for every melanoma patient. In the input file, the number of patients represents the number of columns in the file. The output file contains the prediction outcome with a score. Greater the score, higher is the probability of correct prediction.

#### Data analysis module

This module is used to evaluate the role of each gene in various melanoma states such as regional metastatic, lymph node metastatic and distinct metastatic vs primary tumors based on mRNA and miRNA expression profiles. Moreover, it also incorporates threshold-based MCC of each feature and mean expression values for the RNAseq expression data in the primary and metastatic state of SKCM.

## Discussion and Summary

There is an emergence of synergized clinical and molecular profiles of cancer samples that can aid in predictive modelling for early tumor detection and progression. This prediction helps the physicians in making a suitable decision about the treatment course^66,67^. Previous studies concerning SKCM have focussed on determining its sub types^9^ and survival^27,31^. Li *et al*. made an attempt to predict metastatic progression of melanoma tumor samples and predicted metastatic progression scores using mRNA and miRNA expressions individually; based on which they assigned primary and metastatic samples to primary and metastatic groups. Further, they also found a correlation between clinical characteristics of samples, i.e. Clark’s level and lymph node status with metastatic progression score. Although all of metastatic samples were correctly assigned to the metastatic group; but, many of primary samples were incorrectly assigned to the metastatic group based on the metastatic progression score. They reported that the proportion of runs where a primary tumor specimen was classified as metastatic among the 10,000 runs was also highly non-uniform^38^. But, they did not report the performance of their method in terms of standard parameters (sensitivity, specificity, MCC, Accuracy, AUROC) as well as standard cross-validation techniques have not been implemented (e.g., 10-fold CV, independent validation). Additionally, the classification models were not available to the public. Thus, the current study is an attempt to overcome these inadequacies.

The present study is an effort for the identification of genomic signatures that can classify both metastatic and primary samples with high precision based on mRNA, miRNA and methylation data. Further, our aim is to validate the performance of our prediction model on an independent dataset in addition to 10-fold internal cross validation. Additionally, we have also identified signatures that can further categorize different types of metastatic states, i.e. intra-lymphatic tumor, lymphatic tumor and distant metastatic tumor samples from the primary tumor samples. Furthermore, we also developed a web server to predict and analyse new data based on those identified markers.

In this study, we have identified discriminative genomic features using different feature selection methods and their classification prediction potential elucidated implementing various machine learning algorithms in the segregation of metastatic from primary tumor samples. Our analysis shows that the mRNA expression profile is the strongest predictor of metastasis as compared to miRNA expression and methylation profile. In the current study, the SVC-W model based on the expression of 17 mRNAs is the best performer in discriminating metastatic from primary tumors with overall accuracy of 89.47%, MCC of 0.73 and AUROC 0.95 on independent validation datasets (Table 1). Furthermore, the different models based on mRNA expression were also developed, which can differentiate primary tumors from several states of metastasis with high precision. Interestingly, it has been observed that primary tumor can be easily distinguished from tumors which have metastasized to lymph nodes; as compared to the tumors which have not still metastasized to lymph nodes. Many of the genes from our analysis panel have already been implicated in skin cancer. The *C7* gene has shown to be a potential tumor silencer gene and its expression is highly downregulated in various carcinomas such as ovarian cancer and non-small cell lung cancer (NSCLC)^68^. In current study, this gene alone can correctly predict 83.79% metastatic samples with MCC of 0.56 and AUROC of 0.81. Another gene *MMP3* has been reported to acts as melanoma suppressor gene^69^ and is observed repeatedly in different signatures that distinguish various states of metastatic tumors from primary tumors in our analysis. Further, *KRT14*, a keratin gene, has shown to be downregulated in case of skin cancer^70^. In the present study, it classified metastatic and primary samples with high sensitivity of 94.14% and low specificity of 56.79% with overall the MCC of 0.56. Notably, the role of 11 out of 17 mRNA features have been previously reported in literature for cutaneous melanoma; while 6 genes including *ESM1, NFATC3, C7orf4, CDK14, ZNF827*, and *ZSWIM7* have been described for other cancers and other melanoma types like uveal melanoma but have not been specifically described for cutaneous melanoma^71,72^. Thus, the current study revealed the potential role of these six genes in the classification of the metastatic and primary tumor samples of SKCM for the first time. Earlier, the role of 8 out of 11 genes that include *C7, MMP3, KRT14, KRT17, MASP1, S100A7A, MUC21*, and *DNAJC5B* was previously reported in the metastatic progression of SKCM patients by Li *et al*.^38^. Martins *et al*. observed that one of the genes from our 17-gene signature, *i.e. C10orf12* gets upregulated with the loss of *ColVII* in squamous cell carcinoma model^73^. *CLIC5* which get upregulated in metastatic samples, has been previously shown to be methylated in one of 13 melanoma cell line^74^, while another study elucidated *FILIP1L* as a potential antivascular target for cancer therapy in melanoma model^75^.

Beside mRNA signatures, the miRNA and methylation features were also explored for segregation of primary and metastatic samples. Although these features did not segregate these samples as good as mRNA expression features, we were still able to find that expression of hsa-mir-205 is a strong predictor of metastatic melanoma. In our study, hsa-mir-205 alone can discriminates the metastatic and primary tumors with the sensitivity and specificity of 87.39% and 78.95%, respectively with MCC of 0.61 and AUROC of 0.83, if its expression is less than log2 (RSEM value) of 4.3. The expression of hsa-mir-205 among the miRNAs has shown to be downregulated in various solid tumors^76^. In the recent past, it has been observed that hsa-miR-205 targets oncogenes such as *E2F1* and *E2F5* and downregulates their expression which results in the inhibition of melanoma cell proliferation^24^. In addition, it also acts as tumor suppressor miRNA in skin carcinoma^16,77^, breast cancer^78^ and prostate cancer^79^. We also used average methylation score to segregate the primary and metastatic samples and attained the sensitivity 78.38% and specificity 71.43%with MCC of 0.44 and AUROC of 0.91 with 38 features (Table 5). There is no single gene whose methylation score could segregate metastatic and primary samples well. Also the performance of this model based on 38 features is quite low as compared to the models obtained using gene expression and miRNA expression.

We also developed models combining different omic layers at the feature level (Combo model) and at the model level (ensemble model). Their performance was either less or comparable to the model based on 17 mRNA expression features. Further, we subdivided the heterogeneous group of primary tumors as per the tumor stage and segregated early stage and late stage samples. Based on 37 features, the random forest model segregated these samples with 95.52% sensitivity and 83.87% specificity with MCC of 0.81 and AUROC of 0.96 (Table 7). The features which segregate tumor stages (early and late) is different from features that segregate primary and metastatic samples.

Eventually, we assume that this study would be helpful to recognize important genomic signatures in the classification of primary tumor samples from the metastatic tumor samples of SKCM. Further, our analysis has shown that the genomic features selected by SVC-L1 feature selection method are fewer and have higher performance in classification of the SKCM samples into primary and metastatic classes as compare to the features selected by WEKA-FCBF and PCA methods, respectively. Thus, we hypothesized that this method might prove to be beneficial in scrutinizing important signatures from genomic data for diverse applications. Finally, we have developed the webserver CancerSPP to integrate all the prediction models and tools established in the current study. CancerSPP can analyze the gene expression data of a sample and predict whether it is a primary tumor or metastatic with a score using RSEM values derived from RNAseq and miRNAseq and methylation beta values.

## Material and Methods

### Datasets

The RNAseq, miRNAseq and methylation profiling data for SKCM was retrieved from TCGA project using TCGA - Assembler 2 version^80^. In addition, manifest, biospecimen files and files containing clinical information such as new tumor events, drugs, age, gender, *etc*. were also downloaded to extract clinical parameters using Biospecimen Core Resource (BCR) IDs of patients/subjects. Finally, we obtained 466 patients [102 primary tumor, 74 Regional Cutaneous or Subcutaneous Tissue (includes satellite and in-transit metastasis), 222 Regional Lymph Node and 68 Distant Metastasis samples] for mRNA and methylation data, whereas 444 samples were available [95 primary tumor, 64 Regional Cutaneous or Subcutaneous tissue (includes satellite and in-transit metastasis), 214 Regional Lymph Node and 71 Distant Metastasis] for miRNA expression data. We referred primary tumors, Regional Cutaneous or Subcutaneous Tissue (includes satellite and in-transit metastasis), Regional Lymph Node and Distant Metastasis samples as P1, P2, M1 and M2, respectively. The clinical characteristics of these patients displayed in Supplementary Fig. S5.

In the present study, we have used mRNA and miRNA expression profiles in terms of RSEM values for 20,502 genes and 1,870 miRNAs, respectively. We downloaded the methylation profiles for 20,879 genes (acquired using the Illumina Human-Methylation450K DNA Analysis BeadChip assay, based on genotyping of bisulfite-converted genomic DNA at individual CpG-sites). Notable, we downloaded all CpG sites for each gene, (DNase hypersensitive and non-DNase hypersensitive). The data is in the form of Beta values, a quantitative measure of DNA^81–83^. Here, the methylation value for each gene represents the average of methylation beta values of all CpG sites located on each individual gene.

Further to study the Primary tumors stage-wise analysis using gene expression data, we segregated 67 stage-1 and stage-2 primary SKCM tumors as early stage and 31 stage-3 and stage-4 primary SKCM tumors as late stage SKCM tumors. In case of miRNA, 61 early stage and 30 late stage primary SKCM tumors are available for the analysis.

### Pre-processing of Data

#### Normalization of miRNA and mRNA expression

Z-score Scaling: It has been observed that there is a wide range of variation in RSEM values of mRNAs and miRNAs. Thus, we transformed these values using log2 after addition of 1.0 as a constant number to each of RSEM value. Further, features with low variance were excluded from the data using caret package in R^84^, followed by z-score normalization of data. Thus, log_2_-transformed RSEM values for each mRNA and miRNA were centred and scaled by employing caret package in R. Following equations were used for computing the transformation and normalization:

1 $$x=lo{g}_{2}(RSEM+1)$$

2 $${Z}_{\_score}=\frac{x-\bar{x}}{sd}$$

Where *Z_*_*score*_ is the normalized score, *x* is the log-transformed expression, $\bar{x}$ is the mean of expression and *sd* is the standard deviation of expression.

Min-Max normalization: When we combined all the features from the three omics layers, the RNAseq expression, miRNA expression and methylation profiling data for feature selection in combo model, the Min-Max normalization method in R was employed using range option of preProcess from caret package. This ensured that all three types of features were in the same range of 0 and 1. The validation dataset was transformed in accordance with the training data using predict function in caret.

It was observed that in some of the patients, mRNA expression available for both tissue and blood samples. Here we took the average of both the samples for each patient.

### Feature selection techniques

One of the challenges in developing the prediction model is to extract important features from the large dimension of features. Although, there are a number of methods for feature selection, we have used only those methods, which are well established and previously implemented in similar types of studies^36,37,44–49^. In this study, we implemented three techniques, *i.e*. SVC with L1 penalty employing Scikit package^54^, ‘SymmetricalUncertAttributeSetEval’ with search method of ‘FCBFSearch’ of WEKA software package^85^ and PCA in R. We filtered genes (mRNA expression), methylation pattern of genes and miRNA expression as features that can distinguish metastatic samples from primary tumor samples using these techniques. The FCBF (Fast Correlation-Based Feature) algorithm employed correlation to identify relevant features in high-dimensional datasets in small feature space^39^. SVC-L1 method selects the non-zero coefficients and then applies L1 penalty to select relevant features to reduce dimensions of the data. For feature selection using PCA, we selected those Principal Components that represented at least 1% variance of the data.

To select the robust features, first, the data was split into the ratio of 80:20 for 10 times followed by features selection using SVC-L1 or WEKA-FCBF on each occasion from the training dataset. From this resampling process, we obtained 10 sub-sets of features. We have selected the subset having the highest performance. To check the robustness of features, we computed the average stability index (Jaccard index) using OmicsMarkeR package^86^ for each subset and finally calculated the overall stability index. For all the signatures the average stability index is nearly in the range of 0.40 to 0.43.

### Implementation of machine learning techniques

Firstly, we have developed the prediction models to categorize primary tumor and metastatic samples based on selected genomic features using various classifiers implementing Scikit package. These classifiers include ExtraTrees, KNN, Random forest, Logistic Regression (LR), Ridge classifier and SVC - RBF kernel with class weight factor were implemented employing scikit package. In addition, to understand the progression of skin cancer from primary tumor to metastasis, we also analyse and develop prediction models using various machine learning classifiers of scikit package based on genomic features (mRNA expression) to classify the sub-categories of metastatic samples from primary tumor samples *i.e*. Intra-lymphatic tumors ((P2) v/s primary tumor (P1), lymphatic tumors (M1) v/s primary tumors (P1), distant metastatic tumors (M2) v/s primary tumors (P1), regional (P1P2) v/s lymphatic tumors (M1) and metastatic tumors (M1M2) v/s regional tumor (P1P2).

The optimization of the parameters for the various classifiers was done by using a grid search with area under PR (Precision Recall) curve as scoring performance measure for selecting the best parameter as our data was imbalanced.

### Visualization of samples

After applying supervised learning to classify samples, we visualised the distribution of samples based on selected features on reduced dimensions using t-SNE methods implementing the two R Packages; Rtsne and scatterplot3d packages. t-SNE is a non-linear dimensionality reduction algorithm employed to analyze the high-dimensional data. It converts multi-dimensional data to two or more dimensions^87^.

### Identification of important features using simple threshold-based approach

Here, we employed AUROC and MCC based feature selection technique to identify important features and developed single feature-based prediction models to distinguish metastatic samples from primary tumor samples. Single feature based models are also called threshold based models in which feature having a score below a specific threshold is assigned to metastatic tumor if it is downregulated in metastatic tumor samples otherwise it as primary tumor sample and vice versa. We computed performance of each given feature and identified features having the highest performance in terms of AUROC, MCC with minimum difference in sensitivity and specificity. Additionally, we have also computed their mean difference, log fold change, Bonferroni adjusted p-value using Wilcoxon test in R.

### Performance evaluation of models

In the present study, both internal and independent validation techniques were employed to evaluate the performance of models. Previously, different studies employed the 80:20 ratio for the partitioning of a dataset into training and validation dataset^36,37,88,89^. Therefore, we implemented this standard protocol and subdivided our dataset into two subsets, *i.e*. training dataset and independent validation dataset in ratio of 80:20. We used 80% of the main dataset for training and remaining 20% for independent validation. First, the training dataset is used for developing model and for performing ten-fold cross-validation as internal validation. In this ten-fold-cross validation technique, training dataset is randomly split into ten sets; of which nine out of ten sets are used as training sets and the remaining tenth set as testing dataset. This process is repeated ten times in such a way that each set is exploited once for testing. The final performance of the trained model is the mean performance of all the ten sets.

In order to avoid the over-optimization of parameters in ten-fold cross-validation, we have also implemented independent validation. In the case of independent validation, we evaluated our model on an independent dataset, which was kept aside and remained unseen during feature selection and training or development of the model^90^.

In order to measure the performance of models, we used standard parameters. Both threshold-dependent and threshold-independent parameters were employed to measure the performance. In case of threshold-dependent parameters, we measured sensitivity, specificity, accuracy and Matthews correlation coefficient (MCC) using the following equations.

1 $$Sensitivity\,(Sn)=\frac{TP}{TP+FN}\ast 100$$

2 $$Specificity(Sp)=\frac{TN}{TN+FP}\ast 100$$

3 $$Accuracy\,(Ac)=\frac{TP+TN}{TP+FP+TN+FN}\ast 100$$

4 $$MCC=\frac{(TP\ast TN)-(FP\ast FN)}{\sqrt{TP+FP)(TP+FN)(TN+FP)(TN+FN)}}$$

Where, FP, FN, TP and TN are false positive, false negative, true positive and true negative predictions, respectively.

While, for threshold-independent measures, we used standard parameter AUROC. The AUROC curve is generated by plotting sensitivity or true positive rate against the false positive rate (1-specificity) at various thresholds. Finally, the area under ROC curve was calculated to compute a single parameter called AUROC. Notably, we have obtained classification performance in terms of sensitivity, specificity, accuracy, MCC, AUROC on various thresholds of prediction score for each of prediction models. We have selected only those threshold dependent measures based on the threshold of prediction score that gives maximum accuracy along with the minimum difference between sensitivity and specificity.

### Functional annotation of signature genomic markers

In order to discern the biological relevance of the signature genes, enrichment analysis was performed using Enrichr^91^. Enrichr executes the Fisher exact test to identify enrichment score. It provides Z-score and the adjusted p-value which is derived by applying correction on the Fisher Exact test. Further, to understand the biological impact of miRNAs in metastatic melanoma development, we employed miRTarBase to identify target genes of signature miRNA.

## Supplementary information

Supplementary Information
