# Supplementary material for: Prediction and Analysis of Skin Cancer Progression using Genomics Profiles of Patients
Source: Sci Rep. 2019 Oct 31;9:15790. doi: 10.1038/s41598-019-52134-4 (PMC6823463; doi:10.1038/s41598-019-52134-4)
Supplement: Supplementary file 1 — Supplementary Information [file 41598_2019_52134_MOESM1_ESM.pdf]

# Supplementary Information

## Prediction and analysis of skin cancer progression using genomics profiles of patients

**Sherry Bhalla*[1,2], Harpreet Kaur*[3], Anjali Dhall[1], Gajendra P. S. Raghava[#1]**

**Supplementary Tables:**

Table S1: Performance measures of 17 mRNA expression features based on SVC-RBF-W method at all thresholds for discrimination of metastatic from primary tumor samples of training and independent validation dataset.

| Threshold | Dataset | TP | FP | TN | FN | Sens | Spec | Acc | MCC | AUROC |
|---|---|---|---|---|---|---|---|---|---|---|
| -1 | Training | 288 | 24 | 57 | 2 | 99.31 | 70.37 | 92.99 | 0.79 | 0.97 |
| | Validation | 73 | 10 | 11 | 1 | 98.65 | 52.38 | 88.42 | 0.64 | 0.95 |
| -0.9 | Training | 288 | 22 | 59 | 2 | 99.31 | 72.84 | 93.53 | 0.8 | 0.97 |
| | Validation | 72 | 10 | 11 | 2 | 97.3 | 52.38 | 87.37 | 0.6 | 0.95 |
| -0.8 | Training | 287 | 20 | 61 | 3 | 98.97 | 75.31 | 93.8 | 0.81 | 0.97 |
| | Validation | 71 | 10 | 11 | 3 | 95.95 | 52.38 | 86.32 | 0.57 | 0.95 |
| -0.7 | Training | 286 | 17 | 64 | 4 | 98.62 | 79.01 | 94.34 | 0.83 | 0.97 |
| | Validation | 71 | 8 | 13 | 3 | 95.95 | 61.9 | 88.42 | 0.64 | 0.95 |
| -0.6 | Training | 286 | 15 | 66 | 4 | 98.62 | 81.48 | 94.88 | 0.85 | 0.97 |
| | Validation | 71 | 8 | 13 | 3 | 95.95 | 61.9 | 88.42 | 0.64 | 0.95 |
| -0.5 | Training | 286 | 14 | 67 | 4 | 98.62 | 82.72 | 95.15 | 0.85 | 0.97 |
| | Validation | 71 | 7 | 14 | 3 | 95.95 | 66.67 | 89.47 | 0.68 | 0.95 |
| -0.4 | Training | 283 | 14 | 67 | 7 | 97.59 | 82.72 | 94.34 | 0.83 | 0.97 |
| | Validation | 71 | 7 | 14 | 3 | 95.95 | 66.67 | 89.47 | 0.68 | 0.95 |
| -0.3 | Training | 278 | 14 | 67 | 12 | 95.86 | 82.72 | 92.99 | 0.79 | 0.97 |
| | Validation | 71 | 6 | 15 | 3 | 95.95 | 71.43 | 90.53 | 0.71 | 0.95 |
| -0.2 | Training | 274 | 13 | 68 | 16 | 94.48 | 83.95 | 92.18 | 0.77 | 0.97 |

| | | | | | | | | | | |
|---|---|---|---|---|---|---|---|---|---|---|
| | Validation | 69 | 5 | 16 | 5 | 93.24 | 76.19 | 89.47 | 0.69 | 0.95 |
| -0.1 | Training | 271 | 12 | 69 | 19 | 93.45 | 85.19 | 91.64 | 0.76 | 0.97 |
| | Validation | 69 | 4 | 17 | 5 | 93.24 | 80.95 | 90.53 | 0.73 | 0.95 |
| 0 | Training | 270 | 11 | 70 | 20 | 93.1 | 86.42 | 91.64 | 0.77 | 0.97 |
| | Validation | 67 | 3 | 18 | 7 | 90.54 | 85.71 | 89.47 | 0.72 | 0.95 |
| 0.1 | Training | 269 | 8 | 73 | 21 | 92.76 | 90.12 | 92.18 | 0.79 | 0.97 |
| | Validation | 66 | 2 | 19 | 8 | 89.19 | 90.48 | 89.47 | 0.73 | 0.95 |
| 0.2 | Training | 266 | 7 | 74 | 24 | 91.72 | 91.36 | 91.64 | 0.78 | 0.97 |
| | Validation | 66 | 1 | 20 | 8 | 89.19 | 95.24 | 90.53 | 0.77 | 0.95 |
| 0.3 | Training | 259 | 5 | 76 | 31 | 89.31 | 93.83 | 90.3 | 0.76 | 0.97 |
| | Validation | 65 | 1 | 20 | 9 | 87.84 | 95.24 | 89.47 | 0.75 | 0.95 |
| 0.4 | Training | 255 | 4 | 77 | 35 | 87.93 | 95.06 | 89.49 | 0.75 | 0.97 |
| | Validation | 63 | 1 | 20 | 11 | 85.14 | 95.24 | 87.37 | 0.71 | 0.95 |
| 0.5 | Training | 251 | 4 | 77 | 39 | 86.55 | 95.06 | 88.41 | 0.73 | 0.97 |
| | Validation | 61 | 1 | 20 | 13 | 82.43 | 95.24 | 85.26 | 0.68 | 0.95 |
| 0.6 | Training | 245 | 3 | 78 | 45 | 84.48 | 96.3 | 87.06 | 0.71 | 0.97 |
| | Validation | 59 | 1 | 20 | 15 | 79.73 | 95.24 | 83.16 | 0.64 | 0.95 |
| 0.7 | Training | 240 | 3 | 78 | 50 | 82.76 | 96.3 | 85.71 | 0.69 | 0.97 |
| | Validation | 56 | 1 | 20 | 18 | 75.68 | 95.24 | 80 | 0.6 | 0.95 |
| 0.8 | Training | 229 | 2 | 79 | 61 | 78.97 | 97.53 | 83.02 | 0.65 | 0.97 |
| | Validation | 55 | 1 | 20 | 19 | 74.32 | 95.24 | 78.95 | 0.59 | 0.95 |
| 0.9 | Training | 221 | 2 | 79 | 69 | 76.21 | 97.53 | 80.86 | 0.62 | 0.97 |
| | Validation | 54 | 1 | 20 | 20 | 72.97 | 95.24 | 77.89 | 0.57 | 0.95 |
| 1.0 | Training | 211 | 2 | 79 | 79 | 72.76 | 97.53 | 78.17 | 0.59 | 0.97 |
| | Validation | 53 | 1 | 20 | 21 | 71.62 | 95.24 | 76.84 | 0.56 | 0.95 |

TP: True Positive; FP: False Positive; TN: True Negative; FN: False Negative; Sens: Sensitivity; Spec: Specificity; Acc: Accuracy; MCC: Matthews Correlation Coefficient; AUROC: Area under the Receiver Operating Characteristic

Table S2: Performance measures of 150 mRNA expression-based features (selected using WEKA-FCBF feature selection method) on training and independent validation dataset to classify metastatic from primary samples by applying various machine-learning algorithms.

| Classifier | Dataset | TP | FP | TN | FN | Sens | Spec | Acc | MCC | AUROC |
|---|---|---|---|---|---|---|---|---|---|---|
| **ETrees** | Training | 277 | 10 | 73 | 14 | 95.19 | 87.95 | 93.58 | 0.82 | 0.97 |
| | Validation | 67 | 3 | 17 | 8 | 89.33 | 85 | 88.42 | 0.69 | 0.91 |
| **KNN** | Training | 279 | 13 | 70 | 12 | 95.88 | 84.34 | 93.32 | 0.81 | 0.97 |
| | Validation | 70 | 3 | 17 | 5 | 93.33 | 85 | 91.58 | 0.76 | 0.89 |
| **RF** | Training | 282 | 11 | 72 | 9 | 96.91 | 86.75 | 94.65 | 0.84 | 0.98 |
| | Validation | 69 | 4 | 16 | 6 | 92 | 80 | 89.47 | 0.7 | 0.90 |
| **LR** | Training | 252 | 5 | 78 | 39 | 86.6 | 93.98 | 88.24 | 0.72 | 0.97 |
| | Validation | 63 | 3 | 17 | 12 | 84 | 85 | 84.21 | 0.61 | 0.91 |
| **RC** | Training | 251 | 5 | 78 | 40 | 86.25 | 93.98 | 87.97 | 0.72 | 0.96 |
| | Validation | 66 | 3 | 17 | 9 | 88 | 85 | 87.37 | 0.67 | 0.89 |
| **SVC-W** | Training | 256 | 8 | 75 | 35 | 87.97 | 90.36 | 88.5 | 0.71 | 0.95 |
| | Validation | 63 | 4 | 16 | 12 | 84 | 80 | 83.16 | 0.57 | 0.89 |

Etrees: Extra Trees Classifier; KNN: K-Nearest Neighbors Classifier; RF: Random Forest; LR: Logistic Regression; RC: Ridge Classifier; SVC-W: Support Vector Classification with weight factor; TP: True Positive; FP: False Positive; TN: True Negative; FN: False Negative; Sens: Sensitivity; Spec: Specificity; Acc: Accuracy; MCC: Matthews Correlation Coefficient; AUROC: Area under the Receiver Operating Characteristic

Table S3: Performance measures of 32 Principal Component features from mRNA expression data (selected by using Principal Component Analysis (PCA) for discriminating metastatic from primary samples on training and independent validation dataset by applying various machine-learning algorithms.

| Classifier | Dataset | TP | FP | TN | FN | Sens | Spec | Acc | MCC | AUROC |
|---|---|---|---|---|---|---|---|---|---|---|
| **ETrees** | Training | 200 | 8 | 66 | 77 | 72.2 | 89.19 | 75.78 | 0.51 | 0.90 |
| | Validation | 58 | 5 | 16 | 13 | 81.69 | 76.19 | 80.43 | 0.52 | 0.85 |
| **KNN** | Training | 203 | 16 | 58 | 74 | 73.29 | 78.38 | 74.36 | 0.44 | 0.86 |
| | Validation | 49 | 2 | 19 | 22 | 69.01 | 90.48 | 73.91 | 0.5 | 0.89 |
| **RF** | Training | 223 | 15 | 59 | 54 | 80.51 | 79.73 | 80.34 | 0.53 | 0.87 |
| | Validation | 56 | 6 | 15 | 15 | 78.87 | 71.43 | 77.17 | 0.45 | 0.88 |
| **LR** | Training | 246 | 13 | 61 | 31 | 88.81 | 82.43 | 87.46 | 0.66 | 0.89 |
| | Validation | 66 | 5 | 16 | 5 | 92.96 | 76.19 | 89.13 | 0.69 | 0.91 |
| **RC** | Training | 241 | 13 | 61 | 36 | 87 | 82.43 | 86.04 | 0.63 | 0.89 |
| | Validation | 61 | 5 | 16 | 10 | 85.92 | 76.19 | 83.7 | 0.58 | 0.90 |
| **SVC-W** | Training | 232 | 14 | 60 | 45 | 83.75 | 81.08 | 83.19 | 0.58 | 0.87 |
| | Validation | 59 | 4 | 17 | 12 | 83.1 | 80.95 | 82.61 | 0.58 | 0.90 |

Etrees: Extra Trees Classifier; KNN: K-Nearest Neighbors Classifier; RF: Random Forest; LR: Logistic Regression; RC: Ridge Classifier; SVC-W: Support Vector Classification with weight factor; TP: True Positive; FP: False Positive; TN: True Negative; FN: False Negative; Sens: Sensitivity; Spec: Specificity; Acc: Accuracy; MCC: Matthews Correlation Coefficient; AUROC: Area under the Receiver Operating Characteristic

Table S4: Performance measures of 17 mRNA expression based features (selected using SVC-L1 feature selection method) after removing sixteen samples mapped in NTE file on training and independent validation dataset to classify metastatic from primary samples by applying various machine-learning algorithms.

| **Classifiers** | **Dataset** | **TP** | **FP** | **TN** | **FN** | **Sens** | **Spec** | **Acc** | **MCC** | **AUROC** |
|---|---|---|---|---|---|---|---|---|---|---|
| **ETrees** | Training | 261 | 8 | 60 | 29 | 90 | 88.24 | 89.66 | 0.71 | 0.96 |
| | Validation | 67 | 3 | 15 | 7 | 90.54 | 83.33 | 89.13 | 0.69 | 0.93 |
| **KNN** | Training | 272 | 7 | 61 | 18 | 93.79 | 89.71 | 93.02 | 0.79 | 0.96 |
| | Validation | 68 | 4 | 14 | 6 | 91.89 | 77.78 | 89.13 | 0.67 | 0.93 |
| **RF** | Training | 264 | 5 | 63 | 26 | 91.03 | 92.65 | 91.34 | 0.76 | 0.98 |
| | Validation | 67 | 4 | 14 | 7 | 90.54 | 77.78 | 88.04 | 0.65 | 0.95 |
| **LR** | Training | 262 | 6 | 62 | 28 | 90.34 | 91.18 | 90.5 | 0.74 | 0.98 |
| | Validation | 67 | 2 | 16 | 7 | 90.54 | 88.89 | 90.22 | 0.73 | 0.95 |
| **RC** | Training | 264 | 7 | 61 | 26 | 91.03 | 89.71 | 90.78 | 0.74 | 0.98 |
| | Validation | 65 | 3 | 15 | 9 | 87.84 | 83.33 | 86.96 | 0.64 | 0.95 |
| **SVC-W** | Training | 263 | 4 | 64 | 27 | 90.69 | 94.12 | 91.34 | 0.76 | 0.98 |
| | Validation | 67 | 1 | 17 | 7 | 90.54 | 94.44 | 91.3 | 0.77 | 0.95 |

Etrees: Extra Trees Classifier; KNN: K-Nearest Neighbors Classifier; RF: Random Forest; LR: Logistic Regression; RC: Ridge Classifier; SVC-W: Support Vector Classification with weight factor; TP: True Positive; FP: False Positive; TN: True Negative; FN: False Negative; Sens: Sensitivity; Spec: Specificity; Acc: Accuracy; MCC: Matthews Correlation Coefficient; AUROC: Area under the Receiver Operating Characteristic

Table S5: Performance measures of 10 mRNA expression features (selected using SVC-L1 feature selection method) for discriminating P2 and P1 on training and independent validation dataset by applying various machine-learning algorithms.

| **Classifier** | **Dataset** | **TP** | **FP** | **TN** | **FN** | **Sens** | **Spec** | **Acc** | **MCC** | **AUROC** |
|---|---|---|---|---|---|---|---|---|---|---|
| **ETrees** | Training | 46 | 4 | 77 | 13 | 77.97 | 95.06 | 87.86 | 0.75 | 0.96 |
| | Validation | 10 | 1 | 20 | 5 | 66.67 | 95.24 | 83.33 | 0.66 | 0.85 |
| **KNN** | Training | 50 | 5 | 76 | 9 | 84.75 | 93.83 | 90 | 0.79 | 0.96 |
| | Validation | 11 | 3 | 18 | 4 | 73.33 | 85.71 | 80.56 | 0.60 | 0.84 |
| **RF** | Training | 51 | 6 | 75 | 8 | 86.44 | 92.59 | 90 | 0.79 | 0.97 |
| | Validation | 10 | 2 | 19 | 5 | 66.67 | 90.48 | 80.56 | 0.60 | 0.85 |
| **LR** | Training | 58 | 7 | 74 | 1 | 98.31 | 91.36 | 94.29 | 0.89 | 0.98 |
| | Validation | 11 | 4 | 17 | 4 | 73.33 | 80.95 | 77.78 | 0.54 | 0.86 |
| **RC** | Training | 53 | 8 | 73 | 6 | 89.83 | 90.12 | 90 | 0.80 | 0.97 |
| | Validation | 10 | 5 | 16 | 5 | 66.67 | 76.19 | 72.22 | 0.43 | 0.85 |
| **SVC-W** | Training | 56 | 7 | 74 | 3 | 94.92 | 91.36 | 92.86 | 0.86 | 0.98 |
| | Validation | 11 | 4 | 17 | 4 | 73.33 | 80.95 | 77.78 | 0.54 | 0.86 |

Etrees: Extra Trees Classifier; KNN: K-Nearest Neighbors Classifier; RF: Random Forest; LR: Logistic Regression; RC: Ridge Classifier; SVC-W: Support Vector Classification with weight factor; TP: True Positive; FP: False Positive; TN: True Negative; FN: False Negative; Sens: Sensitivity; Spec: Specificity; Acc: Accuracy; MCC: Matthews Correlation Coefficient; AUROC: Area under the Receiver Operating Characteristic

Table S6: Performance measures of 5 mRNA expression features (selected by using SVC-L1 feature selection method) to discriminate M2 and P1 on training and independent validation dataset by applying various machine-learning algorithms.

| **Classifier** | **Dataset** | **TP** | **FP** | **TN** | **FN** | **Sens** | **Spec** | **Acc** | **MCC** | **AUROC** |
|---|---|---|---|---|---|---|---|---|---|---|
| **ETrees** | Training | 45 | 7 | 74 | 9 | 83.33 | 91.36 | 88.15 | 0.75 | 0.93 |
| | Validation | 11 | 4 | 17 | 3 | 78.57 | 80.95 | 80 | 0.59 | 0.82 |
| **KNN** | Training | 47 | 6 | 75 | 7 | 87.04 | 92.59 | 90.37 | 0.8 | 0.92 |
| | Validation | 11 | 3 | 18 | 3 | 78.57 | 85.71 | 82.86 | 0.64 | 0.81 |
| **RF** | Training | 47 | 9 | 72 | 7 | 87.04 | 88.89 | 88.15 | 0.75 | 0.93 |
| | Validation | 11 | 4 | 17 | 3 | 78.57 | 80.95 | 80 | 0.59 | 0.86 |
| **LR** | Training | 49 | 8 | 73 | 5 | 90.74 | 90.12 | 90.37 | 0.8 | 0.94 |
| | Validation | 11 | 4 | 17 | 3 | 78.57 | 80.95 | 80 | 0.59 | 0.85 |
| **RC** | Training | 50 | 9 | 72 | 4 | 92.59 | 88.89 | 90.37 | 0.8 | 0.94 |
| | Validation | 11 | 5 | 16 | 3 | 78.57 | 76.19 | 77.14 | 0.54 | 0.86 |
| **SVC-W** | Training | 50 | 7 | 74 | 4 | 92.59 | 91.36 | 91.85 | 0.83 | 0.94 |
| | Validation | 11 | 4 | 17 | 3 | 78.57 | 80.95 | 80.00 | 0.59 | 0.85 |

Etrees: Extra Trees Classifier; KNN: K-Nearest Neighbors Classifier; RF: Random Forest; LR: Logistic Regression; RC: Ridge Classifier; SVC-W: Support Vector Classification with weight factor; TP: True Positive; FP: False Positive; TN: True Negative; FN: False Negative; Sens: Sensitivity; Spec: Specificity; Acc: Accuracy; MCC: Matthews Correlation Coefficient; AUROC: Area under the Receiver Operating Characteristic

Table S7: Performance measures of 14 mRNA expression features (selected using SVC-L1 feature selection method) to discriminate M1 and P1_P2 on training and independent validation dataset by applying various machine-learning algorithms.

| Classifier | Dataset | TP | FP | TN | FN | Sens | Spec | Acc | MCC | AUROC |
|---|---|---|---|---|---|---|---|---|---|---|
| **ETrees** | Training | 155 | 20 | 120 | 22 | 87.57 | 85.71 | 86.75 | 0.73 | 0.94 |
| | Validation | 38 | 5 | 31 | 7 | 84.44 | 86.11 | 85.19 | 0.7 | 0.88 |
| **KNN** | Training | 166 | 25 | 115 | 11 | 93.79 | 82.14 | 88.64 | 0.77 | 0.95 |
| | Validation | 40 | 8 | 28 | 5 | 88.89 | 77.78 | 83.95 | 0.67 | 0.89 |
| **RF** | Training | 152 | 18 | 122 | 25 | 85.88 | 87.14 | 86.44 | 0.73 | 0.94 |
| | Validation | 38 | 4 | 32 | 7 | 84.44 | 88.89 | 86.42 | 0.73 | 0.91 |
| **LR** | Training | 163 | 14 | 126 | 14 | 92.09 | 90 | 91.17 | 0.82 | 0.96 |
| | Validation | 42 | 6 | 30 | 3 | 93.33 | 83.33 | 88.89 | 0.78 | 0.89 |
| **RC** | Training | 164 | 18 | 122 | 13 | 92.66 | 87.14 | 90.22 | 0.8 | 0.96 |
| | Validation | 42 | 7 | 29 | 3 | 93.33 | 80.56 | 87.65 | 0.75 | 0.87 |
| **SVC-W** | Training | 160 | 19 | 121 | 17 | 90.4 | 86.43 | 88.64 | 0.77 | 0.96 |
| | Validation | 42 | 7 | 29 | 3 | 93.33 | 80.56 | 87.65 | 0.75 | 0.89 |

Etrees: Extra Trees Classifier; KNN: K-Nearest Neighbors Classifier; RF: Random Forest; LR: Logistic Regression; RC: Ridge Classifier; SVC-W: Support Vector Classification with weight factor; TP: True Positive; FP: False Positive; TN: True Negative; FN: False Negative; Sens: Sensitivity; Spec: Specificity; Acc: Accuracy; MCC: Matthews Correlation Coefficient; AUROC: Area under the Receiver Operating Characteristic

Table S8: Performance measures of 15 mRNA expression features (selected by using SVC-L1 feature selection method) to discriminate M1_M2 vs P1_P2 on training and independent validation dataset by applying various machine-learning algorithms.

| Classifier | Dataset | TP | FP | TN | FN | Sens | Spec | Acc | MCC | AUROC |
|---|---|---|---|---|---|---|---|---|---|---|
| ETrees | Training | 184 | 18 | 122 | 47 | 79.65 | 87.14 | 82.48 | 0.65 | 0.90 |
| | Validation | 44 | 4 | 32 | 15 | 74.58 | 88.89 | 80 | 0.62 | 0.90 |
| KNN | Training | 207 | 34 | 106 | 24 | 89.61 | 75.71 | 84.37 | 0.66 | 0.90 |
| | Validation | 49 | 7 | 29 | 10 | 83.05 | 80.56 | 82.11 | 0.63 | 0.88 |
| RF | Training | 185 | 18 | 122 | 46 | 80.09 | 87.14 | 82.75 | 0.65 | 0.91 |
| | Validation | 42 | 4 | 32 | 17 | 71.19 | 88.89 | 77.89 | 0.58 | 0.90 |
| LR | Training | 207 | 22 | 118 | 24 | 89.61 | 84.29 | 87.6 | 0.74 | 0.93 |
| | Validation | 48 | 7 | 29 | 11 | 81.36 | 80.56 | 81.05 | 0.61 | 0.90 |
| RC | Training | 204 | 26 | 114 | 27 | 88.31 | 81.43 | 85.71 | 0.7 | 0.93 |
| | Validation | 48 | 7 | 29 | 11 | 81.36 | 80.56 | 81.05 | 0.61 | 0.89 |
| SVC-W | Training | 201 | 24 | 116 | 30 | 87.01 | 82.86 | 85.44 | 0.69 | 0.93 |
| | Validation | 46 | 6 | 30 | 13 | 77.97 | 83.33 | 80 | 0.6 | 0.91 |

Etrees: Extra Trees Classifier; KNN: K-Nearest Neighbors Classifier; RF: Random Forest; LR: Logistic Regression; RC: Ridge Classifier; SVC-W: Support Vector Classification with weight factor; TP: True Positive; FP: False Positive; TN: True Negative; FN: False Negative; Sens: Sensitivity; Spec: Specificity; Acc: Accuracy; MCC: Matthews Correlation Coefficient; AUROC: Area under the Receiver Operating Characteristic

Table S9: Performance measures of 43 Principal Component features from miRNA expression data (selected by using Principal Component Analysis (PCA) for discriminating metastatic from primary samples on training and independent validation dataset by applying various machine-learning algorithms.

| **Classifier** | **Dataset** | **TP** | **FP** | **TN** | **FN** | **Sens** | **Spec** | **Acc** | **MCC** | **AUROC** |
|---|---|---|---|---|---|---|---|---|---|---|
| **ETrees** | Training | 186 | 12 | 62 | 91 | 67.15 | 83.78 | 70.66 | 0.42 | 0.87 |
| | Validation | 53 | 8 | 13 | 18 | 74.65 | 61.9 | 71.74 | 0.32 | 0.81 |
| **KNN** | Training | 247 | 24 | 50 | 30 | 89.17 | 67.57 | 84.62 | 0.55 | 0.86 |
| | Validation | 59 | 6 | 15 | 12 | 83.1 | 71.43 | 80.43 | 0.5 | 0.79 |
| **RF** | Training | 212 | 16 | 58 | 65 | 76.53 | 78.38 | 76.92 | 0.47 | 0.88 |
| | Validation | 50 | 9 | 12 | 21 | 70.42 | 57.14 | 67.39 | 0.24 | 0.77 |
| **LR** | Training | 222 | 15 | 59 | 55 | 80.14 | 79.73 | 80.06 | 0.52 | 0.87 |
| | Validation | 59 | 5 | 16 | 12 | 83.1 | 76.19 | 81.52 | 0.54 | 0.85 |
| **RC** | Training | 224 | 13 | 61 | 53 | 80.87 | 82.43 | 81.2 | 0.55 | 0.88 |
| | Validation | 59 | 5 | 16 | 12 | 83.1 | 76.19 | 81.52 | 0.54 | 0.86 |
| **SVC-W** | Training | 221 | 16 | 58 | 56 | 79.78 | 78.38 | 79.49 | 0.51 | 0.87 |
| | Validation | 56 | 5 | 16 | 15 | 78.87 | 76.19 | 78.26 | 0.49 | 0.83 |

Etrees: Extra Trees Classifier; KNN: K-Nearest Neighbors Classifier; RF: Random Forest; LR: Logistic Regression; RC: Ridge Classifier; SVC-W: Support Vector Classification with weight factor; TP: True Positive; FP: False Positive; TN: True Negative; FN: False Negative; Sens: Sensitivity; Spec: Specificity; Acc: Accuracy; MCC: Matthews Correlation Coefficient; AUROC: Area under the Receiver Operating Characteristic

Table S10: Performance measures of 25 Principal Component features from methylation data (selected by using Principal Component Analysis (PCA) for discriminating metastatic from primary samples on training and independent validation dataset by applying various machine-learning algorithms.

| **Classifier** | **Dataset** | **TP** | **FP** | **TN** | **FN** | **Sens** | **Spec** | **Acc** | **MCC** | **AUROC** |
|---|---|---|---|---|---|---|---|---|---|---|
| **ETrees** | Training | 157 | 17 | 57 | 120 | 56.68 | 77.03 | 60.97 | 0.27 | 0.79 |
| | Validation | 36 | 5 | 16 | 35 | 50.7 | 76.19 | 56.52 | 0.23 | 0.65 |
| **KNN** | Training | 215 | 39 | 35 | 62 | 77.62 | 47.3 | 71.23 | 0.23 | 0.72 |
| | Validation | 52 | 11 | 10 | 19 | 73.24 | 47.62 | 67.39 | 0.19 | 0.63 |
| **RF** | Training | 179 | 17 | 57 | 98 | 64.62 | 77.03 | 67.24 | 0.34 | 0.78 |
| | Validation | 36 | 6 | 15 | 35 | 50.7 | 71.43 | 55.43 | 0.19 | 0.67 |
| **LR** | Training | 208 | 22 | 52 | 69 | 75.09 | 70.27 | 74.07 | 0.39 | 0.79 |
| | Validation | 48 | 10 | 11 | 23 | 67.61 | 52.38 | 64.13 | 0.17 | 0.68 |
| **RC** | Training | 185 | 15 | 59 | 92 | 66.79 | 79.73 | 69.52 | 0.38 | 0.79 |
| | Validation | 42 | 7 | 14 | 29 | 59.15 | 66.67 | 60.87 | 0.22 | 0.69 |
| **SVC-W** | Training | 202 | 19 | 55 | 75 | 72.92 | 74.32 | 73.22 | 0.4 | 0.79 |
| | Validation | 51 | 9 | 12 | 20 | 71.83 | 57.14 | 68.48 | 0.26 | 0.70 |

Etrees: Extra Trees Classifier; KNN: K-Nearest Neighbors Classifier; RF: Random Forest; LR: Logistic Regression; RC: Ridge Classifier; SVC-W: Support Vector Classification with weight factor; TP: True Positive; FP: False Positive; TN: True Negative; FN: False Negative; Sens: Sensitivity; Spec: Specificity; Acc: Accuracy; MCC: Matthews Correlation Coefficient; AUROC: Area under the Receiver Operating Characteristic

Table S11: Performance measures of 17 mRNA expression features and one miRNA (combo or hybrid) in discriminating metastatic from primary tumor samples on training and independent validation dataset by applying various machine-learning algorithms.

| Classifier | Dataset | TP | FP | TN | FN | Sens | Spec | Acc | MCC | AUROC |
|---|---|---|---|---|---|---|---|---|---|---|
| **ETrees** | Training | 262 | 11 | 65 | 16 | 94.24 | 85.53 | 92.37 | 0.78 | 0.96 |
| | Validation | 65 | 5 | 14 | 5 | 92.86 | 73.68 | 88.76 | 0.67 | 0.95 |
| **KNN** | Training | 259 | 8 | 68 | 19 | 93.17 | 89.47 | 92.37 | 0.79 | 0.96 |
| | Validation | 63 | 2 | 17 | 7 | 90 | 89.47 | 89.89 | 0.73 | 0.91 |
| **RF** | Training | 264 | 9 | 67 | 14 | 94.96 | 88.16 | 93.5 | 0.81 | 0.96 |
| | Validation | 63 | 3 | 16 | 7 | 90 | 84.21 | 88.76 | 0.69 | 0.93 |
| **LR** | Training | 256 | 5 | 71 | 22 | 92.09 | 93.42 | 92.37 | 0.8 | 0.97 |
| | Validation | 62 | 2 | 17 | 8 | 88.57 | 89.47 | 88.76 | 0.71 | 0.94 |
| **RC** | Training | 252 | 5 | 71 | 26 | 90.65 | 93.42 | 91.24 | 0.77 | 0.97 |
| | Validation | 62 | 3 | 16 | 8 | 88.57 | 84.21 | 87.64 | 0.67 | 0.93 |
| **SVC-W** | Training | 269 | 12 | 64 | 9 | 96.76 | 84.21 | 94.07 | 0.82 | 0.97 |
| | Validation | 66 | 4 | 15 | 4 | 94.29 | 78.95 | 91.01 | 0.73 | 0.93 |

Etrees: Extra Trees Classifier; KNN: K-Nearest Neighbors Classifier; RF: Random Forest; LR: Logistic Regression; RC: Ridge Classifier; SVC-W: Support Vector Classification with weight factor; TP: True Positive; FP: False Positive; TN: True Negative; FN: False Negative; Sens: Sensitivity; Spec: Specificity; Acc: Accuracy; MCC: Matthews Correlation Coefficient; AUROC: Area under the Receiver Operating Characteristic

Table S12: Distribution of 20 features selected from combined 3 types of genomic data (RNAseq, miRNAseq, methylation data) by SVC-L1 feature selection method to build hybrid model.

| Type of Data | Name or Gene symbol |
|---|---|
| mRNA or Gene | *C10orf12, C7, DNAJC5B, EPHX4, ESM1, FABP4, GLRB, KRT14, MARCO, MMP3, OR2L2, S100A7, SCN4A, SGK196* |
| miRNA | hsa-mir-205 |
| Methylation of Genes | *SGIP1, FDPS, ERLEC1, ZIC1, MB* |

Table S13: Performance measures of 20 hybrid features (14 mRNA + 1 miRNA + 5 methylation features) selected using SVC-L1 feature selection method from after combining RNAseq, miRNAseq and methylation-seq data to discriminate metastatic from primary samples on training and independent validation dataset by applying various machine-learning algorithms.

| **Classifier** | **Dataset** | **TP** | **FP** | **TN** | **FN** | **Sens** | **Spec** | **Acc** | **MCC** | **AUROC** |
|---|---|---|---|---|---|---|---|---|---|---|
| **ETrees** | Training | 252 | 5 | 69 | 25 | 90.97 | 93.24 | 91.45 | 0.78 | 0.97 |
| | Validation | 63 | 4 | 17 | 8 | 88.73 | 80.95 | 86.96 | 0.66 | 0.91 |
| **KNN** | Training | 242 | 5 | 69 | 35 | 87.36 | 93.24 | 88.6 | 0.72 | 0.96 |
| | Validation | 63 | 4 | 17 | 8 | 88.73 | 80.95 | 86.96 | 0.66 | 0.91 |
| **RF** | Training | 248 | 8 | 66 | 29 | 89.53 | 89.19 | 89.46 | 0.72 | 0.96 |
| | Validation | 67 | 3 | 18 | 4 | 94.37 | 85.71 | 92.39 | 0.79 | 0.91 |

| | | | | | | | | | | |
|---|---|---|---|---|---|---|---|---|---|---|
| **LR** | Training | 271 | 13 | 61 | 6 | 97.83 | 82.43 | 94.59 | 0.83 | 0.98 |
| | Validation | 70 | 6 | 15 | 1 | 98.59 | 71.43 | 92.39 | 0.78 | 0.90 |
| **RC** | Training | 221 | 2 | 72 | 56 | 79.78 | 97.3 | 83.48 | 0.65 | 0.97 |
| | Validation | 58 | 3 | 18 | 13 | 81.69 | 85.71 | 82.61 | 0.6 | 0.91 |
| **SVC-W** | Training | 238 | 7 | 67 | 39 | 85.92 | 90.54 | 86.89 | 0.68 | 0.95 |
| | Validation | 56 | 4 | 17 | 15 | 78.87 | 80.95 | 79.35 | 0.53 | 0.89 |

Etrees: Extra Trees Classifier; KNN: K-Nearest Neighbors Classifier; RF: Random Forest; LR: Logistic Regression; RC: Ridge Classifier; SVC-W: Support Vector Classification with weight factor; TP: True Positive; FP: False Positive; TN: True Negative; FN: False Negative; Sens: Sensitivity; Spec: Specificity; Acc: Accuracy; MCC: Matthews Correlation Coefficient; AUROC: Area under the Receiver Operating Characteristic (AUROC) curve

Table S14: Important miRNAs and top 20 genes with their performance measures selected on the basis of a simple expression threshold based method.

| **Gene symbol/ miRNA** | **Thresh** | **Sens** | **spec** | **Acc** | **MCC** | **AUROC** | **Mean_diff (M-P)** | **LogFC** | **Bonferroni adjusted p-value** | **Regulation status in metastatic samples** |
|---|---|---|---|---|---|---|---|---|---|---|
| hsa-mir-205 | 4.3 | 87.39 | 78.95 | 85.59 | 0.61 | 0.83 | -5.89 | 2.11 | 1.31E-25 | Downregulated |
| *C7* | 4.3 | 83.79 | 77.78 | 82.48 | 0.56 | 0.81 | 4.46 | 1.26 | 6.77E-27 | Upregulated |
| *S100A7* | 3.1 | 90 | 66.67 | 84.91 | 0.56 | 0.78 | -6.27 | 2.77 | 1.21E-14 | Downregulated |
| *LOC642587* | 0.9 | 85.17 | 69.14 | 81.67 | 0.51 | 0.77 | -3.32 | 2.89 | 2.22E-12 | Downregulated |
| *CASP14* | 0.9 | 94.14 | 59.26 | 86.52 | 0.58 | 0.77 | -3.81 | 3.91 | 1.86E-10 | Downregulated |

| | | | | | | | | | | |
|---|---|---|---|---|---|---|---|---|---|---|
| *MMP3* | 3.7 | 73.79 | 80.25 | 75.2 | 0.46 | 0.77 | -3.59 | 1.30 | 9.11E-19 | Downregulated |
| *ANXA8L2* | 1.9 | 87.93 | 60.49 | 81.94 | 0.48 | 0.76 | -3.81 | 2.49 | 1.51E-12 | Downregulated |
| *KRTDAP* | 4 | 95.86 | 56.79 | 87.33 | 0.6 | 0.76 | -5.33 | 3.22 | 4.78E-13 | Downregulated |
| *DSG1* | 3.2 | 89.66 | 62.96 | 83.83 | 0.53 | 0.76 | -4.95 | 2.35 | 1.76E-13 | Downregulated |
| *CLCA2* | 4.9 | 87.24 | 64.2 | 82.21 | 0.5 | 0.76 | -4.24 | 1.42 | 1.21E-14 | Downregulated |
| *TRIM29* | 3.9 | 83.1 | 67.9 | 79.78 | 0.47 | 0.76 | -4.97 | 1.63 | 4.28E-14 | Downregulated |
| *WFDC5* | 2 | 96.21 | 55.56 | 87.33 | 0.6 | 0.76 | -3.55 | 3.73 | 2.36E-11 | Downregulated |
| hsa-mir-203b | 1 | 91.69 | 60 | 84.91 | 0.54 | 0.75 | -1.73 | 2.54 | 4.70E-14 | Downregulated |
| *ODZ2* | 4 | 87.59 | 61.73 | 81.94 | 0.48 | 0.75 | -2.98 | 1.19 | 1.95E-12 | Downregulated |
| *KRT14* | 9.1 | 94.14 | 56.79 | 85.98 | 0.56 | 0.75 | -7.38 | 1.68 | 6.82E-19 | Downregulated |
| *KLK5* | 3 | 94.83 | 56.79 | 86.52 | 0.58 | 0.75 | -4.81 | 3.02 | 1.30E-13 | Downregulated |
| *LGALS7B* | 3 | 93.79 | 55.56 | 85.44 | 0.54 | 0.75 | -4.59 | 2.87 | 2.70E-12 | Downregulated |
| *IRX4* | 2 | 92.41 | 56.79 | 84.64 | 0.53 | 0.75 | -2.34 | 2.23 | 5.81E-11 | Downregulated |
| *LCE3D* | 2 | 94.14 | 55.56 | 85.71 | 0.55 | 0.75 | -3.87 | 3.24 | 1.21E-10 | Downregulated |
| *TNS4* | 5.1 | 93.79 | 51.85 | 84.64 | 0.51 | 0.73 | -3.17 | 1.13 | 1.64E-10 | Downregulated |
| *PRSS8* | 4 | 87.93 | 55.56 | 80.86 | 0.44 | 0.72 | -2.99 | 1.22 | 1.14E-10 | Downregulated |
| *GGT6* | 1.2 | 83.1 | 61.73 | 78.44 | 0.42 | 0.72 | -2.71 | 2.09 | 1.87E-09 | Downregulated |

Thresh: Threshold for Expression values of gene or miRNA in terms of Log2 (RSEM) values; TP: True Positive; FP: False Positive; TN: True Negative; FN: False Negative; Sens: Sensitivity; Spec: Specificity; Acc: Accuracy; MCC: Matthews Correlation Coefficient; AUROC: Area under the Receiver Operating Characteristic (AUROC) curve; Mean_diff (M-P): Difference in the mean expression of specific gene/miRNA in metastatic tumor samples and mean expression of specific gene/miRNA in primary tumor samples; LogFC: Log Fold Change (metastatic to primary tumor samples);

**Supplementary Figures:**

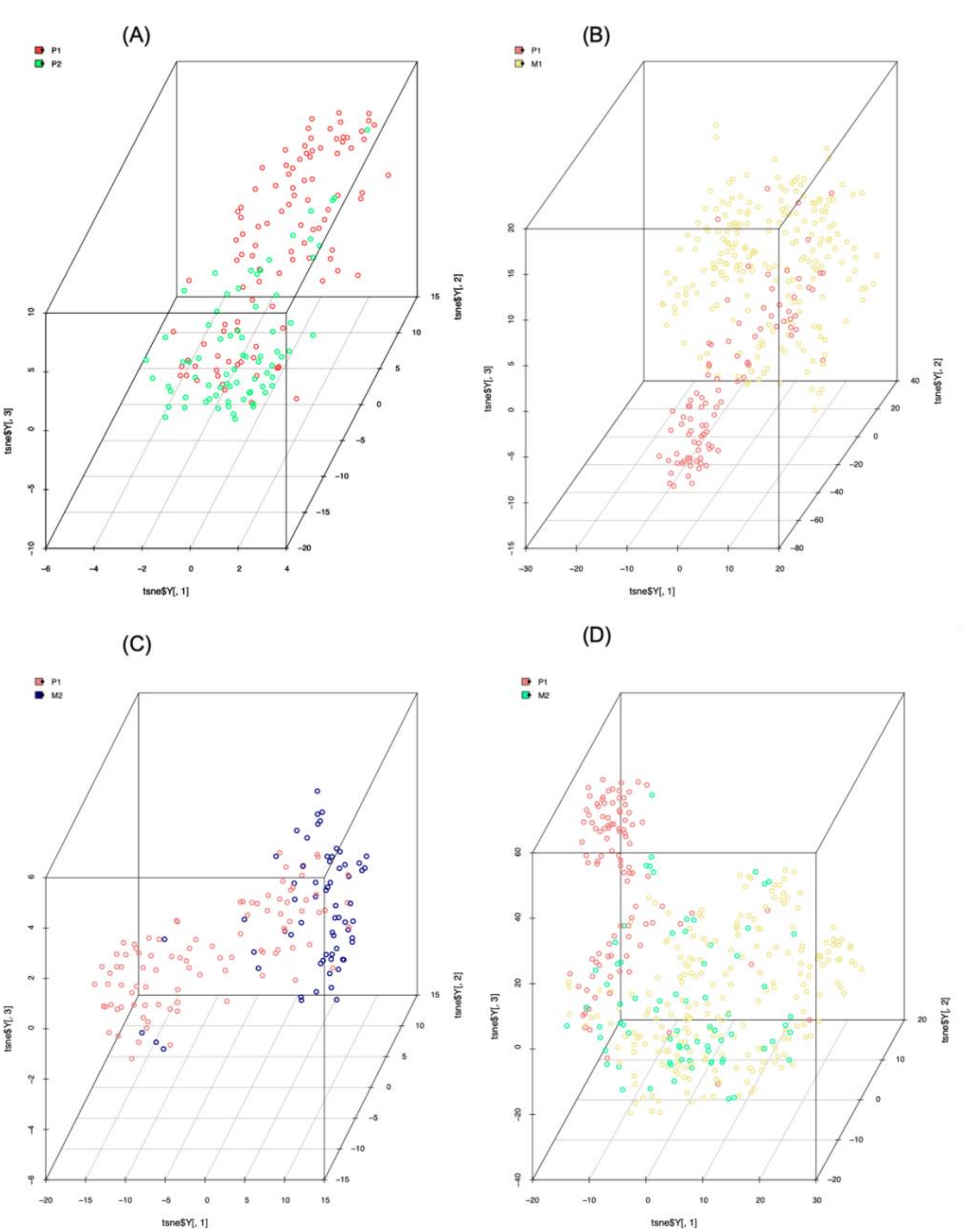


Figure S1: The scatterplot3D view of tSNE dimension reduction of 17 selected features: (A) distribution of P1 and P2 samples on 17 features; (B) distribution of P1 and M1 samples; (C) distribution of P1 and M2 samples; (D) distribution of primary tumors (P1) in comparison to in-transit and satellite tumors (P2) from lymphatic metastatic tumors (M1).

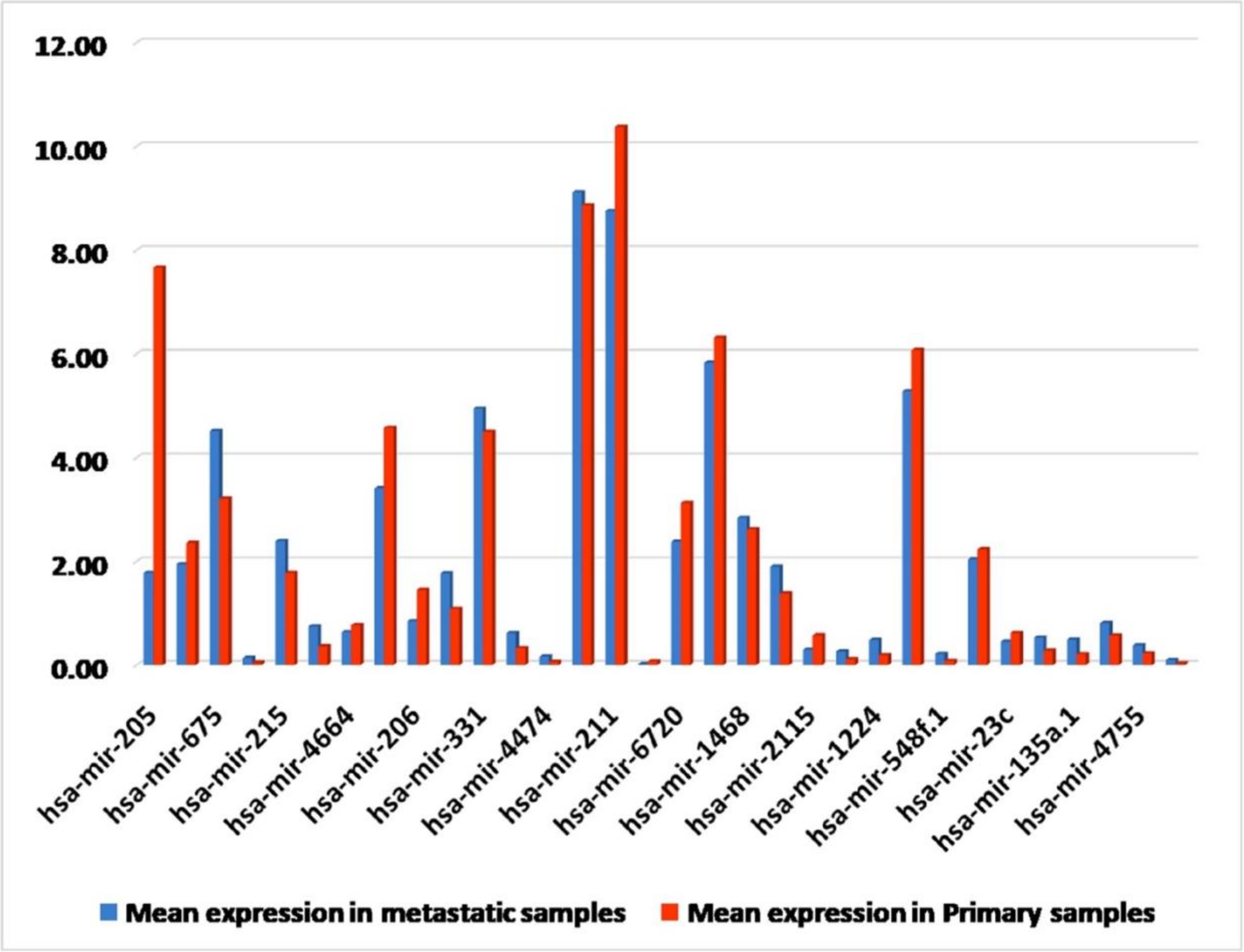


Figure S2: The average expression pattern of WEKA-FCBF selected miRNA features in primary and metastatic tumors.

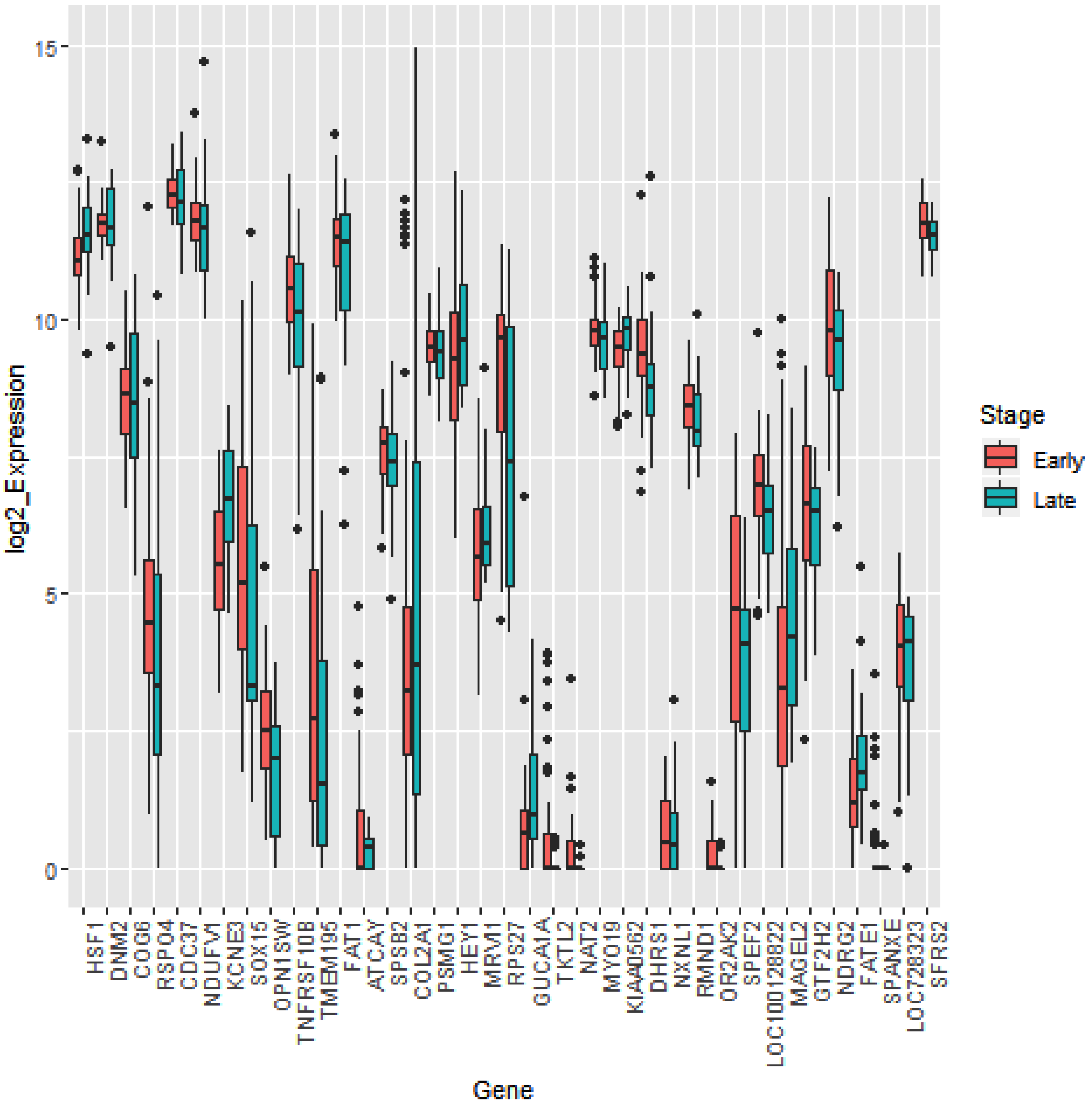


Figure S3: The 37 mRNA expression signature selected using WEKA-FCBF to distinguish primary early stage and primary late stage samples.

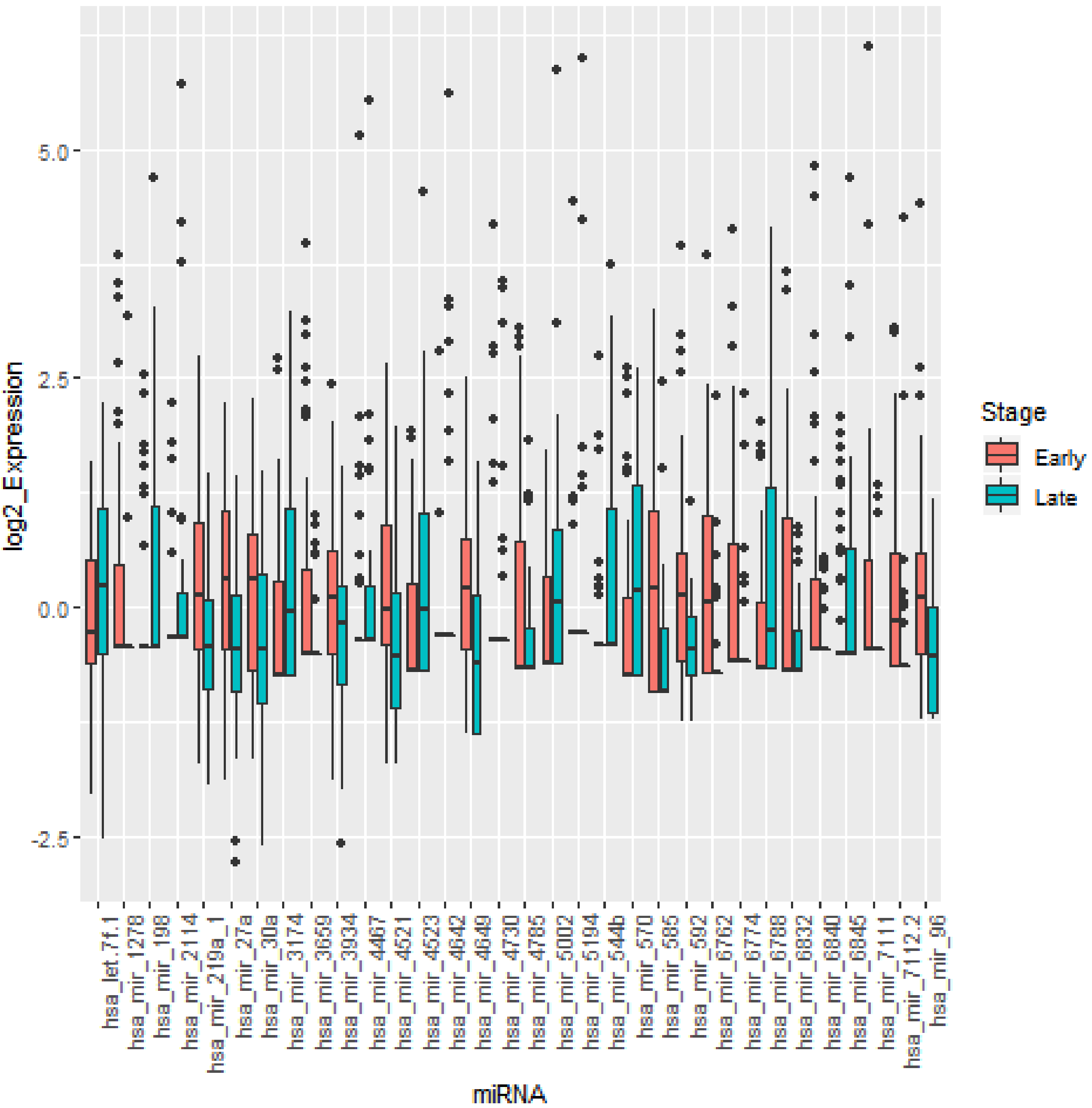


Figure S4: The 32 miRNA expression signature selected using SVC-L1 to distinguish primary early stage and primary late stage samples.

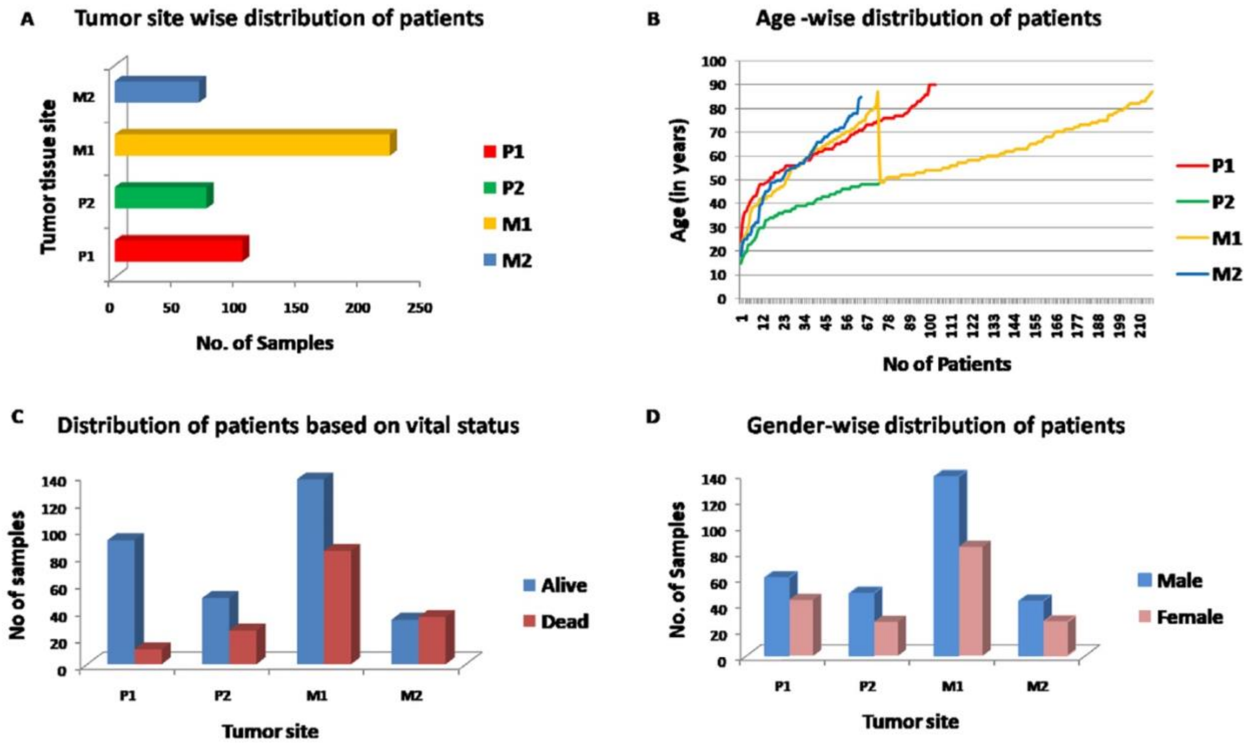


Figure S5: Clinical characteristics of patients used in this study: (A) represents distribution tissue sites in patients. (B) Age wise distribution of patients (C) Distribution of tumor sites based on the vital status (D) Gender wise distribution of tumor sites.
